# Development and validation of a LAMP-based method for rapid and reliable detection of *Xanthomonas albilineans*, the causal agent of sugarcane leaf scald

**DOI:** 10.3389/fmicb.2025.1537812

**Published:** 2025-01-30

**Authors:** Moutoshi Chakraborty, Shamsul Arafin Bhuiyan, Simon Strachan, Muhammad J. A. Shiddiky, Nam-Trung Nguyen, Rebecca Ford

**Affiliations:** ^1^Centre for Planetary Health and Food Security (CPHFS), Griffith University (GU), Brisbane, QLD, Australia; ^2^School of Environment and Science (ESC), Griffith University (GU), Brisbane, QLD, Australia; ^3^Sugar Research Australia (SRA), Brisbane, QLD, Australia; ^4^Queensland Micro- and Nanotechnology Centre (QMNC), Griffith University (GU), Brisbane, QLD, Australia; ^5^Rural Health Research Institute (RHRI), Charles Sturt University, Bathurst, NSW, Australia

**Keywords:** nucleic acid isolation, plant pathogen diagnostic, LS disease, *Xanthomonas albilineans*, rapid detection

## Abstract

**Introduction:**

*Xanthomonas albilineans* (Xalb)-induced leaf scald (LS) is a significant bacterial disease affecting sugarcane and posing a global threat to the sugarcane industry. The presence of irregular symptoms makes traditional phenotypic detection difficult, and molecular methods necessitate costly equipment, labor, and extended sample-to-answer processing times.

**Methods:**

This study introduces an innovative rapid DNA isolation method requiring no reagents, combined with an isothermal amplification-based assay for efficient detection of *Xalb* DNA in sugarcane xylem sap, leaf tissue, and meristematic tissue samples. Sugarcane samples from infected plants were subjected to heat lysis, followed by loop-mediated isothermal amplification (LAMP)-based fluorescence and colorimetric quantification within a single microcentrifuge tube.

**Results:**

The method exhibited exceptional detection sensitivity (detecting as low as 1 cell/μL), reproducibility [with a standard deviation (SD) of <5% for *n* = 3], and a broad linear dynamic range (10 pM to 1 aM or 10^7^–10^0^ copies/μL, *r* = 0.99). Quantification of *Xalb* was accurately correlated with sugarcane cultivar disease ratings. Validation using qPCR showed 91–98% agreement. This assay also effectively determined optimal sampling times and plant parts by monitoring the progression of the disease over time.

**Discussion:**

This diagnostic assay holds significant potential as a commercial opportunity for a kit-based DNA extraction/purification-free molecular detection alternative. It can be adapted into a handheld device, enabling on-farm detection and quantification of the pathogen responsible for LS disease.

## 1 Introduction

Sugarcane holds significant importance as a global crop, serving as a vital source of sugar and biofuel for millions of people. However, its production faces a serious and widespread threat from disease, with leaf scald (LS) being one of the most important. Caused by the xylem-inhabiting bacterium *Xanthomonas albilineans* (Ashby) Dowson (*Xalb*) (Garces et al., 2014), LS has been reported in over 60 countries. Particularly affected are Australia, United States, Philippines, Vietnam, and Thailand (Rott and Davis, 2000). The origins of LS in Australia can be traced back to 1911, with subsequent spread across all sugarcane-growing regions (Rott and Davis, 1995; Davis et al., 1997). The LS disease impact may be severe, leading to significant yield losses, reduced juice quality, and in some cases, the complete loss of entire sugarcane fields (Ricaud and Ryan, 1989; Rott and Davis, 2000; Ntambo et al., 2019). The consequences of LS’s presence are so pronounced that approximately 20% of potentially high-yielding sugarcane cultivars in Australia are rejected due to their susceptibility to this disease (Birch, 2001). This alarming situation has prompted the recognition of LS as a top-priority pest threat in the national plant biosecurity status report. As researchers and authorities work to find solutions, preserving the health and productivity of sugarcane crops remains of utmost importance (Plant Health Australia, 2019).

*Xalb* infects the xylem vessels of sugarcane plants, giving rise to distinctive white-yellow pencil lines along the primary leaf veins. In severe cases, this bacterial infection leads to chlorosis, necrosis, and even the death of the entire plant (Rott and Davis, 2000; Lin et al., 2018). The pathogen is known to produce a potent pathotoxin called albicidin, which acts as a DNA gyrase inhibitor, causing foliar symptoms by hindering the replication of chloroplast DNA (Birch and Patil, 1985). Additionally, albicidin is believed to facilitate systemic invasion and potentially trigger the transition from a latent to an active disease state (Cociancich et al., 2015). Interestingly, many sugarcane cultivars can tolerate the presence of the pathogen without displaying any visible symptoms, or the symptoms may be so mild that they go unnoticed (Ricaud and Ryan, 1989; Rott and Davis, 1995; Rott et al., 1997). Unfortunately, this latent infection can lead to the inadvertent distribution of infected seed cane across the sugarcane production regions. Given these complexities, accurately detecting *Xalb* is crucial for effective LS diagnosis and management. Therefore, it is important to develop methodologies that offer high sensitivity and specificity in quantitative *Xalb* detection.

Traditionally, the diagnosis of LS disease and the assessment of sugarcane test cultivars for LS resistance have relied on observing phenotypic symptoms. However, the latent and erratic nature of symptom expression makes visual disease diagnosis challenging and can lead to the mislabeling of susceptible cultivars as “resistant.” This difficulty in accurately identifying infected plants has been a significant contributor to the global spread of LS through seemingly “healthy” planting materials (Rott, 1995). To address these challenges, various alternative approaches have been explored for LS diagnosis. These methods include isolation on selective media (Davis et al., 1994), microscopy (Mensi et al., 2014), ELISA (enzyme-linked immunosorbent assay) (Wang et al., 1999), PCR (polymerase chain reaction) (Dias et al., 2018), and qPCR (quantitative polymerase chain reaction) (Garces et al., 2014). However, each of these techniques has its limitations. Isolation on selective media and microscopy can be highly effective, especially for detecting *Xalb* in symptomless plants, but is a cumbersome and time-consuming process (Wang et al., 1999; Mensi et al., 2014). Immunological and molecular methods offer varying degrees of sensitivity for detecting *Xalb*, but their wide application has been limited due to the need for complex equipment and advanced laboratory facilities, and reliance on expensive commercial kits or complex chemical-based laborious and time-consuming DNA extraction/purification procedures (Pan et al., 1999; Garces et al., 2014). These factors present obstacles in developing an on-farm rapid *Xalb* detection method and underscore the need for a straightforward, rapid, and cost-effective *Xalb* detection method that minimizes or eliminates the need for chemical reagents in sample preparation.

In this study, we present a streamlined method for the rapid isolation and *in-situ* quantification of *Xalb* DNA. This approach leverages a direct heat lysis approach to release DNA from infected sugarcane samples, avoiding the need for costly commercial kits or complex reagents. This reagent-free DNA isolation method, combined with loop-mediated isothermal amplification (LAMP), offers a simple, rapid solution for *Xalb* detection and quantification. LAMP is known for its efficiency, low cost, and effectiveness in detecting plant pathogens even in crude extracts, making it more resilient than PCR-based methods, which may be affected by amplification inhibitors (Tsai et al., 2009; Francois et al., 2011). Additionally, the method provides both qualitative detection (visual or naked-eye assessment) and quantitative measurement of *Xalb* DNA.

Therefore, this study thus aimed to: (i) develop a simplified, LAMP-based diagnostic method paired with a rapid, reagent-free DNA isolation technique for *in-situ Xalb* detection and quantification from various sugarcane samples; (ii) validate the diagnostic’s quantitative accuracy through qPCR; (iii) assess for correlation of this two-stage diagnostic approach with previously established LS ratings across sugarcane cultivars with known resistance; and (iv) identify optimal sampling times and plant parts for application of the developed diagnostic by tracking disease progression over time.

## 2 Materials and methods

### 2.1 Reagents and materials

All reagents and chemicals used were of analytical grade. Nuclease-free water from Integrated DNA Technologies, Australia, was employed for preparing all aqueous solutions. The PureLink^™^ Microbiome DNA purification kit was purchased from Thermo Fisher Scientific, Australia. Additionally, all designed primers and the synthetic target (4.2 × 10^7^ copies/μL) were purchased from Integrated DNA Technologies, United States. The WarmStart^®^ Colorimetric LAMP 2X Master Mix and WarmStart^®^ Multi-Purpose RT-LAMP 2X Master Mix, as well as the 2X SensiFAST SYBER No-ROX Master Mix, were obtained from New England Biolabs, Ipswich, MA, United States, and Meridian Bioscience, Cincinnati, Ohio, United States, respectively. Furthermore, all other reagents were purchased from Sigma-Aldrich, United States.

### 2.2 Source of bacteria and culture conditions

The *Xalb* strain 3/14/9 bacterium was obtained from the Sugar Research Australia (SRA), Indooroopilly Research Station, Indooroopilly, Queensland, Australia (S 27.50°, E 152.98°). It was then cultured in the modified liquid broth Wilbrink’s media at a temperature of 28°C for 5 days, following the protocol of Davis et al. (1994). *Leifsonia xyli* subsp. *xyli* (*Lxx*), the bacterium responsible for sugarcane ratoon stunting disease (RSD), was used as a negative control. *Lxx* was isolated from an infected sugarcane plant at the SRA Woodford Pathology Research Station (S 26.93°, E 152.78°) in Queensland, Australia. It was cultured in a modified liquid S8 medium and incubated at 28°C for 4 weeks with gentle shaking at 200 rpm in the dark, following the protocols of Davis et al. (1980) and Chakraborty et al. (2024).

### 2.3 Field trial establishment, inoculation, and planting

Three separate field trials with the same cultivars were conducted at the SRA Pathology Research Station in Woodford (S 26.93°, E 152.78°) for this study from 2022–2023. For this, 10 sugarcane cultivars were obtained from a disease-free propagation block at the same research facility, which was established using sugarcane setts treated with long hot water (50°C for 2 h) with a sterile cane knife to eliminate systemic *Xalb* infections (Table 1). Two-month-old plants were inoculated with *Xalb* 2–3 months before sampling using the protocol of Koike (1965). On a cloudy day, the cane tops of six plants per cultivar were decapitated using sharp short, handled cane knives above the growing point between the 3rd and 4th dewlap. Subsequently, the *Xalb* bacteria were “painted” onto the cut tissue with a brush dipped in the inoculum (~10^8^ cells/mL). Each replicated plot comprises 3 m (length) × 1.5 m (width) with a 1 m gap between two plots. The soil preparation for the trial involved maintaining good soil structure (red and grey soils) and ensuring moisture conservation through minimum tillage practices. The region receives an annual rainfall of 1,200–1,400 mm, supporting optimal soil conditions for the experiment. The trials were established using a randomized complete block (RCB) design, with three replications, each consisting of six plants.

**Table 1:** List of sugarcane cultivars used and their LS disease ratings, categorized by phenotypic symptoms (Rott et al., 1997).

Cultivar	LS rating	Rating category
Q68	1	Resistant
Q208	1	Resistant
Q124	2	Resistant
Q133	4	Intermediate resistant
Q96	5	Intermediate resistant
Q63	6	Intermediate susceptible
Q87	7	Susceptible
Q189	8	Highly susceptible
Q44	9	Highly susceptible
Q199	9	Highly susceptible

### 2.4 Sugarcane field sample collection

Sugarcane leaf tissues, meristematic tissues, and xylem sap were sampled from three stalks (one stalk/replication of each cultivar) at different time points of post-inoculation from the SRA Woodford LS field trial in July 2022, October 2022, and January 2023 for the first trial. For xylem sap, vascular extracts (2 mL/cultivar) were obtained using positive air pressure extraction, as outlined by Croft et al. (1994). The extracts were kept on ice during transport to the laboratory and subsequently stored at −20°C until further processing. To validate the assay’s performance using field-derived samples, sugarcane samples were collected from eight cultivars (Q68, Q208, Q124, Q133, Q96, Q63, Q87, and Q44) with various disease ratings (Table 1) initially included in the first trial. In subsequent trials, two additional highly susceptible cultivars (Q189 and Q199) were included to obtain more consistent results (Table 1).

To determine the best leaf position for detecting *Xalb* presence, leaf tissues were collected from three locations on each of the three plants across all 10 sugarcane cultivars (Table 1). These positions were chosen based on their relevance to *Xalb* detection, ensuring consistent and reliable sampling. Leaf tissue samples were collected monthly from the second LS trial between May 2023 and December 2023 to observe consistency at different time points. Samples were taken from the top visible dewlap (TVD) leaf, the topmost fully expanded leaf with a visible dewlap, as well as from TVD +1 and TVD −1, the leaves immediately above and below the TVD leaf, respectively. These three positions served as standard reference points for consistent sampling. To track *Xalb* population development in the leaf tissues, xylem sap, and meristematic tissues of the highly susceptible (Q44) and resistant (Q208) cultivars over time, samples were collected monthly from October 2022 to October 2023 from the third LS field trial at SRA Woodford following the same protocols described above. LS disease resistance was assessed over multiple years based on visible symptoms for most of the cultivars (Rott et al., 1997), as detailed in Table 1.

### 2.5 DNA extraction

Genomic DNA from cultured *Xalb* cells and LS-infected sugarcane samples was extracted for qPCR analysis using the protocol provided in The PureLink^™^ Microbiome DNA purification kit manual (Thermo Fisher Scientific, Australia). Each experiment was performed in triplicate with three replications to ensure consistency.

For the detection of the *Xalb* bacteria via LAMP assay, a rapid, reagent-free method for DNA isolation was utilized, as detailed by Chakraborty et al. (2024). The concentration of bacteria was estimated subsequently via comparison against a standard curve of known *Xalb* amounts and consequential LAMP detection limitation was determined. To establish the standard curve, a series of titrated cultured *Xalb* cells (ranging from 10^7^ to 10^0^ cells/μL) was introduced into disease-free sugarcane xylem sap samples (100 μL). These sap samples were collected from SRA’s disease-free propagation block, confirmed as *Xalb*-free by qPCR, and then subjected to boiling at 95°C for 2 min in a heat block. Subsequently, 2 μL of the boiled suspension was pipetted directly into LAMP or qPCR reactions. Each concentration was replicated three times.

Following the establishment of standard detection curves, field collected leaf tissue and meristematic tissue samples were cut into small pieces and incubated in 100 μL of distilled water for 10 min. The sample solution was then heat lysed by boiling at 95°C for 2 min in a heat block, and 2 μL of the suspension was used for further analysis. Similarly, for the xylem sap samples, 100 μL of sap was directly boiled at 95°C for 2 min without any incubation, and 2 μL of the suspension was used for analysis. Each experiment was conducted three times to ensure accuracy and reliability.

### 2.6 Target selection and primer design

Primers were designed to target a specific 196 bp region within the *XALB1* albicidin pathotoxin biosynthesis gene cluster between positions 43,128 and 43,323 of *Xanthomonas albilineans* (GenBank accession no. AJ586576.1) (National Center for Biotechnology Information, 1988). The selection was based on the crucial role that albicidin plays in the pathogenesis of *Xalb*, as previously highlighted (Royer et al., 2004; Pieretti et al., 2009). Additionally, this region was not homologous to any other species sequenced to date in the GenBank database.

**Figure 1:**
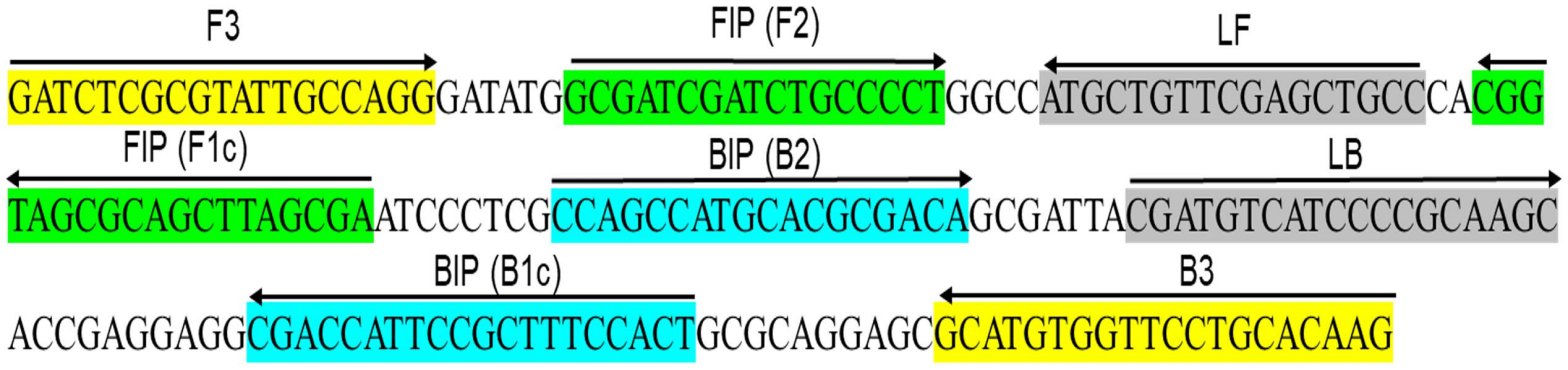
Schematic illustration depicts the primer design for the proposed LAMP assay, showing the locations of the eight primers that span the *Xanthomonas albilineans* gene cluster *XALB1* sequence (GenBank Accession No. AJ586576.1). The right arrow indicates the sense sequence of the primer, while the left arrow represents its complementary sequence.

For LAMP analysis, six primers were designed to recognize a total of eight distinct regions within the target sequence (Table 2 and Figure 1). These included two loop primers (*Xalb*LF and *Xalb*LP), two outer primers (*Xalb*F3 and *Xalb*B3), and two inner primers (*Xalb*FIP and *Xalb*BIP). The design process was carried out using the NEB LAMP primer designing tool available at https://lamp.neb.com. For qPCR analysis, forward and reverse primers (*Xalb*FP and *Xalb*RP) (Table 2) were designed using the NCBI primer blast web tool (Ye et al., 2012). To ensure the specificity of each primer, the corresponding sequences were screened against the NCBI nucleotide and genome databases using the BLASTn tool (Chen et al., 2015). All primer sequences showed 100% homology to the corresponding *Xalb* sequences. Furthermore, the possibility of hairpin and dimer formation was assessed using the OligoAnalyzer^™^ Tool provided by Integrated DNA Technologies Inc., United States to ensure the primer design robustness and avoid any potential issues with primer self-interactions.

**Table 2:** Details of primer sets designed to develop *Xalb*-specific LAMP & qPCR assay.

Synthetic target and primer name	Sequence (5′–3′)	Length (nt)	GC (%)
*Xalb*STS	GATCTCGCGTATTGCCAGGGATATGGCGATCGATCTGCCCCTGGCCA TGCTGTTCGAGCTGCCCACGGTAGCGCAGCTTAGCGAATCCCTCGC CAGCCATGCACGCGACAGCGATTACGATGTCATCCCCGCAAGCACC GAGGAGGCGACCATTCCGCTTTCCACTGCGCAGGAGCGCATGTGG TTCCTGCACAAG	196	61.2
*Xalb*F3	GATCTCGCGTATTGCCAGG	19	57.9
*Xalb*B3	CTTGTGCAGGAACCACATGC	20	55
*Xalb*FIP (F1c-F2)	TCGCTAAGCTGCGCTACCG-GCGATCGATCTGCCCCT	19–17	63.2–64.7
*Xalb*BIP (B2-B1c)	CCAGCCATGCACGCGACA-AGTGGAAAGCGGAATGGTCG	18–20	66.7–55
*Xalb*LF	GGCAGCTCGAACAGCAT	17	58.8
*Xalb*LB	CGATGTCATCCCCGCAAGC	19	63.2
*Xalb*FP	GATCTCGCGTATTGCCAGG	19	57.9
*Xalb*RP	CTTGTGCAGGAACCACATGC	20	55

### 2.7 LAMP reaction conditions

The standard LAMP mixture conditions were followed (Yang et al., 2022), with slight adjustments following the manufacturer’s instructions. For WarmStart fluorescent LAMP/RT-LAMP reactions, a 25 μL mixture was prepared, consisting of 2.5 μL of a 10X LAMP primer concentration (2 μM each of *Xalb*F3 and *Xalb*B3, 16 μM each of *Xalb*FIP and *Xalb*BIP, and 4 μM each of *Xalb*LF and *Xalb*LB). To this, 12.5 μL of 2X WarmStart Multi-purpose master mix, 0.5 μL of SYBR Green fluorescent dye, 2 μL of DNA template, and 7.5 μL of nuclease-free water were added. For the fluorescent LAMP assay, real-time amplification was performed at 65°C for 40 min using a CFX96 Touch Real-Time PCR Detection System (Bio-Rad Laboratories Pty Ltd., Australia). In this LAMP assay, fluorescence threshold time (Tt) is used to quantify amplification, analogous to Cq (quantification cycle) in qPCR. Unlike qPCR, LAMP amplification is isothermal and proceeds continuously, yet Tt represents a measurable and reproducible point where fluorescence exceeds a defined threshold (Schmidt et al., 2021). For WarmStart colorimetric LAMP reactions, a 25-μL mixture was prepared, comprising 2.5 μL of a 10X LAMP primer concentration (2 μM each of *Xalb*F3 and *Xalb*B3, 16 μM each of *Xalb*FIP and *Xalb*BIP, and 4 μM each of *Xalb*LF and *Xalb*LB). To this, 12.5 μL of 2X WarmStart colorimetric LAMP master mix, 2 μL of DNA template, and 8 μL of nuclease-free water were added. The mixture was incubated at 65°C for 40 min using the CFX96 Touch Real-Time PCR Detection System (Bio-Rad Laboratories Pty Ltd., Australia). Subsequently, the amplified products were stored at 4°C prior to further analysis. The colorimetric LAMP products were visually inspected, with samples turning yellow classified as *Xalb*-positive, and those remaining pink considered *Xalb*-negative, following the manufacturer’s instructions (New England Biolabs, United States). Sterile distilled water (ddH_2_O) or fresh sap were used as no-target controls (NTCs). Meanwhile, *Leifsonia xyli* subsp. *xyli* (*Lxx*) cells or purified DNA from *Lxx* cells were utilized as negative controls (NG). Each sample, NTC or NG was analyzed in triplicate to evaluate intra-assay variability which was determined by calculating the SD (standard deviation) between the Tt (threshold time) values for each replicate.

### 2.8 Validation of qPCR assay and gel documentation

The qPCR experiments were conducted following the guidelines provided by the manufacturer (New England Biolabs, United States). Each qPCR mixture, with a volume of 20 μL, consisted of 10 μL of 2X SensiFAST SYBER No-ROX Mix, 0.8 μL each of *Xalb*FP (10 μM) and *Xalb*RP (10 μM), 2 μL of DNA template, and 6.4 μL of nuclease-free water. To perform the qPCR, a CFX96 Touch Real-Time PCR Detection System (Bio-Rad Laboratories Pty Ltd., Australia) was utilized, applying the following reaction conditions: initial denaturation at 100°C for 1 min, 40 cycles at 98°C for 15 s, 52°C for 30 s, and 72°C for 30 s. The reaction was then terminated with a heating step at 72°C for 2 min, followed by a hold at 4°C for 5 min. At the completion of the reaction, the Cq value for each dilution was analyzed. A positive result for *Xalb* was recognized if it was observed within less than 40 cycles. Each assay was performed in triplicate for each repetition to ensure accuracy and reliability. For gel documentation, 5 μL of each LAMP or PCR product was loaded onto a 1% agarose gel and electrophoresed in 1X Tris-acetate-EDTA (TAE) buffer at 90 V for 40 min. The gel was then stained with SYBR Safe and visualized using UV light on a gel documentation system. The sizes of the amplified products were estimated using a 100 bp GeneRuler (Thermo Fisher Scientific, Australia).

### 2.9 Statistical analysis

Data analyses were conducted using OriginPro 2022 v.9.9.0.225 (OriginLab, Northampton, Massachusetts, United States), the R programming language (version 4.2.1), and Microsoft Excel 365 (United States). To analyze the sensitivity, serial dilutions of known *Xalb* target sequence, *Xalb* cells, and purified genomic DNA concentrations were utilized to create a standard curve for the absolute quantification of *Xalb*. This standard curve was represented as a semi-log regression line plot, with Tt and Cq values plotted against the (−log) of the input DNA template amount. The efficiency (*E*) of the fluorescence LAMP and qPCR assays was calculated using the formula *E* = (10^−1/slope^) − 1. Both the fluorescence LAMP and qPCR results obtained with the target-specific primers were validated within the range of 0.9 < *E* < 1.1, with an *E* value closer to 1.0 indicating higher amplification efficiency. To estimate the development of bacterial population over time in leaf tissue, meristematic tissue, and xylem sap of two sugarcane cultivars (Q44 and Q208) using LAMP, a logistic model was used as:


$$P=\frac{d}{1+e^{b\left(t-c\right)}}$$

where, *P* is estimated development of *Xalb* population, *d* is estimated maximum population, *b* is estimated rate of development, *c* is inflection point or estimated median *Xalb* population development time, and *t* is *Xalb* population development time (month).

To convert the LAMP data (Tt values) to *Xalb* population or Cells the following equation was utilized:


$$x=\frac{a+b}{y}$$

where, *x* is the logarithm of the initial cell number *a* is the y-intercept, *b* is slope of the standard curve obtained from the *Xalb* cell number, and *y* is the Tt value.

To predict the cultivar disease ratings for *Xalb*, Tt and Cq values from the analysis of sugarcane samples were considered, whereby a correlation between Cq values and pathogen titer, was previously shown to correspond to disease ratings of cultivars (Davis et al., 1988; Rott et al., 1997). To analyze the Tt and Cq datasets, a linear mixed model was fitted using proc mixed in SAS version 9.4 (SAS Institute, Cary, NC). This model enabled the evaluation and interpretation of Tt and Cq data in relation to the disease ratings of sugarcane cultivars. Cultivars were considered as fixed effects, while block (replication) and the error term (residual) were regarded as random effects in the statistical analysis. To determine appropriate significance factors, protected-mean comparisons of all possible pairwise differences in Tt and Cq values were analyzed at alpha = 0.05 using Fisher’s protected LSD test. The PDMIX800 SAS Macro was employed to convert the mean separation outputs into letter groupings (Saxton, 1998). Spearman rank correlation was conducted to assess the relationship and accuracy of the newly developed method by comparing the outputs of the LAMP and qPCR techniques. Data visualizations were created using BioRender (BioRender, 2022), SnapGene software,^1^ and Microsoft PowerPoint 365 (United States). The color change observed in the colorimetric LAMP reaction was captured using a mobile camera (Samsung Galaxy A52s).

## 3 Results

### 3.1 Validation of novel *Xalb* target sequences and assessment of LAMP assay robustness

The newly designed LAMP primer sets successfully amplified the targeted *XALB1* albicidin pathotoxin biosynthesis gene cluster region (196 bp) in both colorimetric and fluorescent LAMP assays using 10 pM (equivalent to 10^7^ copies/μL) of synthetic target sequences. This confirmed the efficacy of the primers in detecting *Xalb* (Supplementary Figure S1A). In the colorimetric LAMP assay, a distinct color change from pink to yellow indicated a positive response when *Xalb* targets were present, while the no-template control (NTC) and negative control (NC) remained pink (Supplementary Figure S1B), highlighting the specificity of the primers and the suitability of the assay for naked-eye detection of *Xalb*. In the gel electrophoresis, the LAMP amplification products exhibited a typical ladder-like pattern, with the primary band corresponding to the expected 196 bp amplicon, along with other bands resulting from the amplification properties of LAMP (Supplementary Figure S1C).

**Figure 2:**
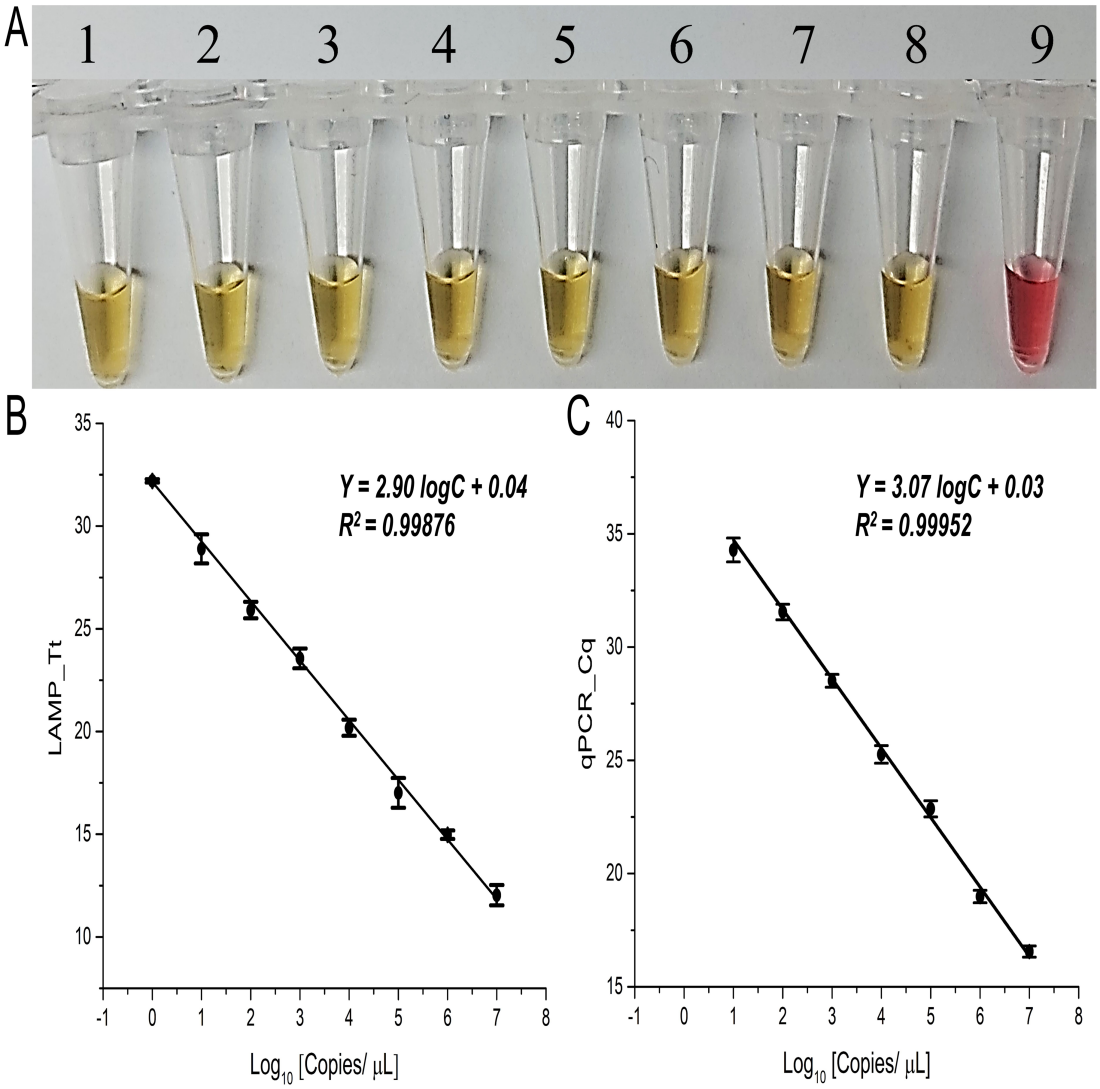
Validation of LAMP and qPCR primers with synthetic target concentrations. **(A)** Colorimetric LAMP detection for the specified concentrations; tubes 1 to 8 show 1:10 dilutions of the synthetic target (10^7^–10^0^ copies/μL, or 10 pg/μL–1 ag/μL); tube 9 is the no target control (NTC). **(B)** Fluorescent LAMP Tt values and **(C)** qPCR Cq values for the specified synthetic target concentrations (10^7^–10^0^ copies/μL, or 10 pg/μL–1 ag/μL). Error bars indicate the standard deviation from three biological replicates.

Furthermore, the positive color change to yellow was consistently observed across all eight serially diluted concentrations of synthetic target sequences, from 10^7^ to 10^0^ copies/μL, with the lowest detection limit of this assay of 1 copy/μL (1 ag/μL) (Figure 2A). Similarly, in the fluorescent LAMP assay, detection of the synthetic targets was achieved solely in the infected sap samples, with no signal observed in the NTC (Supplementary Figure S2). The fluorescent LAMP signal exhibited a direct and reproducible correlation with the target DNA concentration, as indicated by the Tt values obtained at various time points throughout the reaction (Figure 2B), yielding a high correlation coefficient (*r*) of 0.99. The lowest detection limit for the LAMP assay remained at 1 copy/μL (1 ag/μL), and gel electrophoresis confirmed the amplification of the expected 196 bp product, along with additional bands, exclusively in positive reactions (Figures 2A; Supplementary Figure S2).

To further validate the results of the LAMP assay, a standard curve for qPCR was established using titrated synthetic targets ranging from 10^7^ to 10^0^ copies/μL of *Xalb* (Figure 2C), with a lowest concentration detected was 10 copies/μL (100 ag/μL) or 1 nM at an annealing temperature of 52°C (Supplementary Figure S5A), which was 10 times less sensitive than the LAMP assay (Figure 2B). Moreover, a single amplicon of the correct size (196 bp) was visualized exclusively from the positive samples (Supplementary Figures S5B, S6B), further supporting the robustness and reliability of the LAMP assay for detecting *Xalb*.

### 3.2 Validation of LAMP-based *in-situ* detection and quantification method for *Xalb*

**Figure 3:**
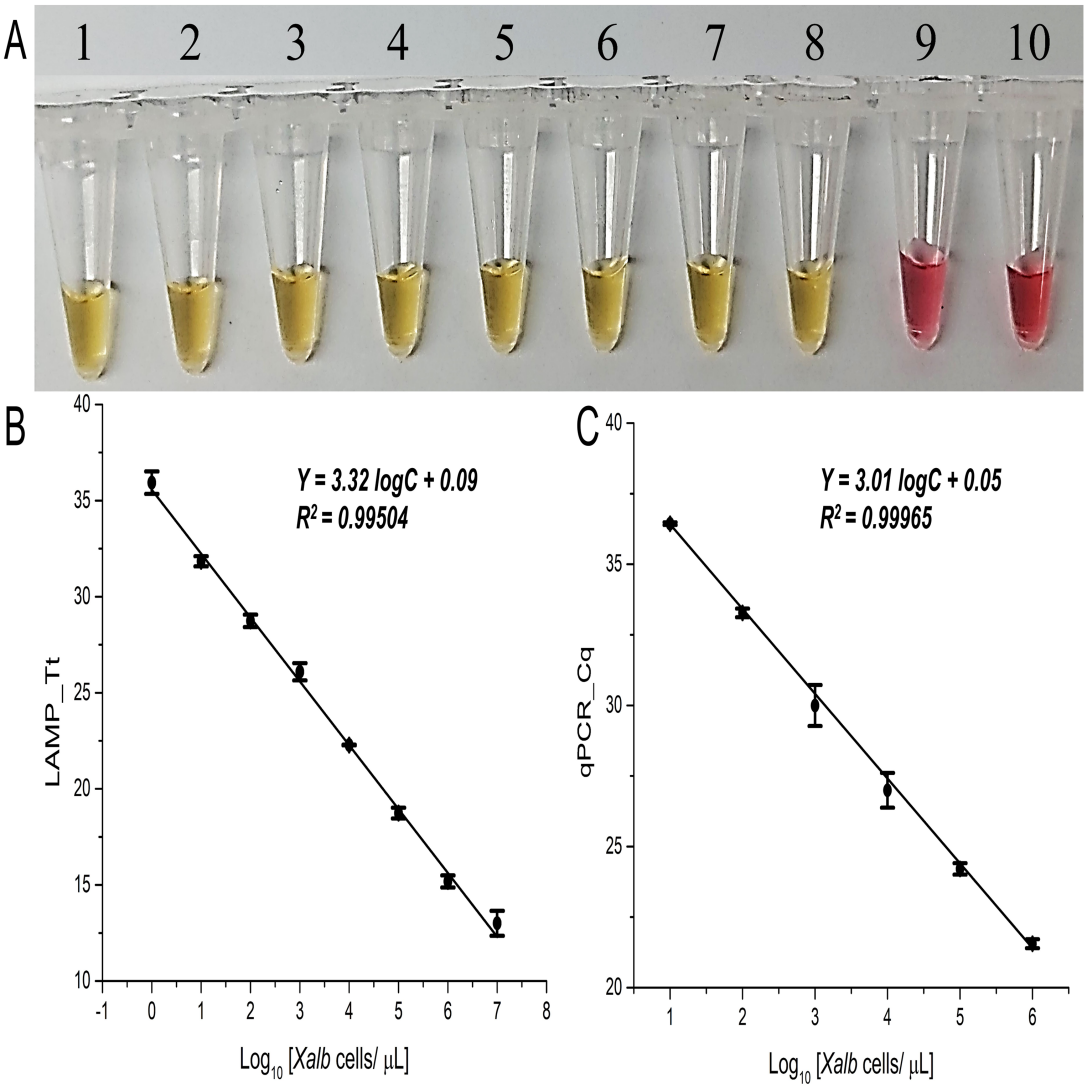
Sensitivity and specificity analysis of the LAMP and qPCR assays. **(A)** Colorimetric LAMP detection for the specified samples; Tubes 1 to 8 contain 1:10 dilutions with *Xalb* cells spiked into fresh, clean sap (10_7_–10_0_ cells/μL); Tube 9 is the No Target Control (NTC); Tube 10 contains a known concentration of spiked *Lxx* cells (10_7_ cells/µL). **(B)** Tt values from fluorescence LAMP, and **(C)** Cq values from qPCR detection for the specified concentrations of Xalb cells spiked into fresh xylem sap (10_7_–10_0_ cells/µL). Error bars indicate the standard deviation across three biological replicates.

Using the standard curve generated from predetermined titrated concentrations of *Xalb* cells (10^7^–10^0^ cells/μL), the lowest detection limit of the colorimetric LAMP assay was approximately 1 cell/μL (Figure 3A), consistent with the fluorescence LAMP assay results (SD ≤5% across three replicates, *r* = 0.99; Figure 3B). Additionally, the expected LAMP amplicon of 196 bp, along with a ladder-like pattern, was visible on agarose gel for positive samples only (Supplementary Figure S3).

To further validate the LAMP assay with *Xalb* cell samples, a qPCR standard curve was created by plotting Cq values against the log-transformed quantities of purified DNA extracted from titrated *Xalb* cell concentrations. The qPCR assay reliably amplified the target region from as few as 100 *Xalb* cells (Figures 3C; Supplementary Figure S6B), with the expected 196 bp amplicon observed on agarose gel for positive samples (Supplementary Figure S6D). A strong correlation (*r* = 0.99) was observed between Tt and Cq values from LAMP and qPCR assays. Notably, the LAMP assay showed a sensitivity 100 times greater than qPCR for *in-situ* detection and quantification of *Xalb* bacteria (Figure 3B vs. Figure 3C).

### 3.3 Detection of *Xalb* in different sugarcane samples

**Figure 4:**
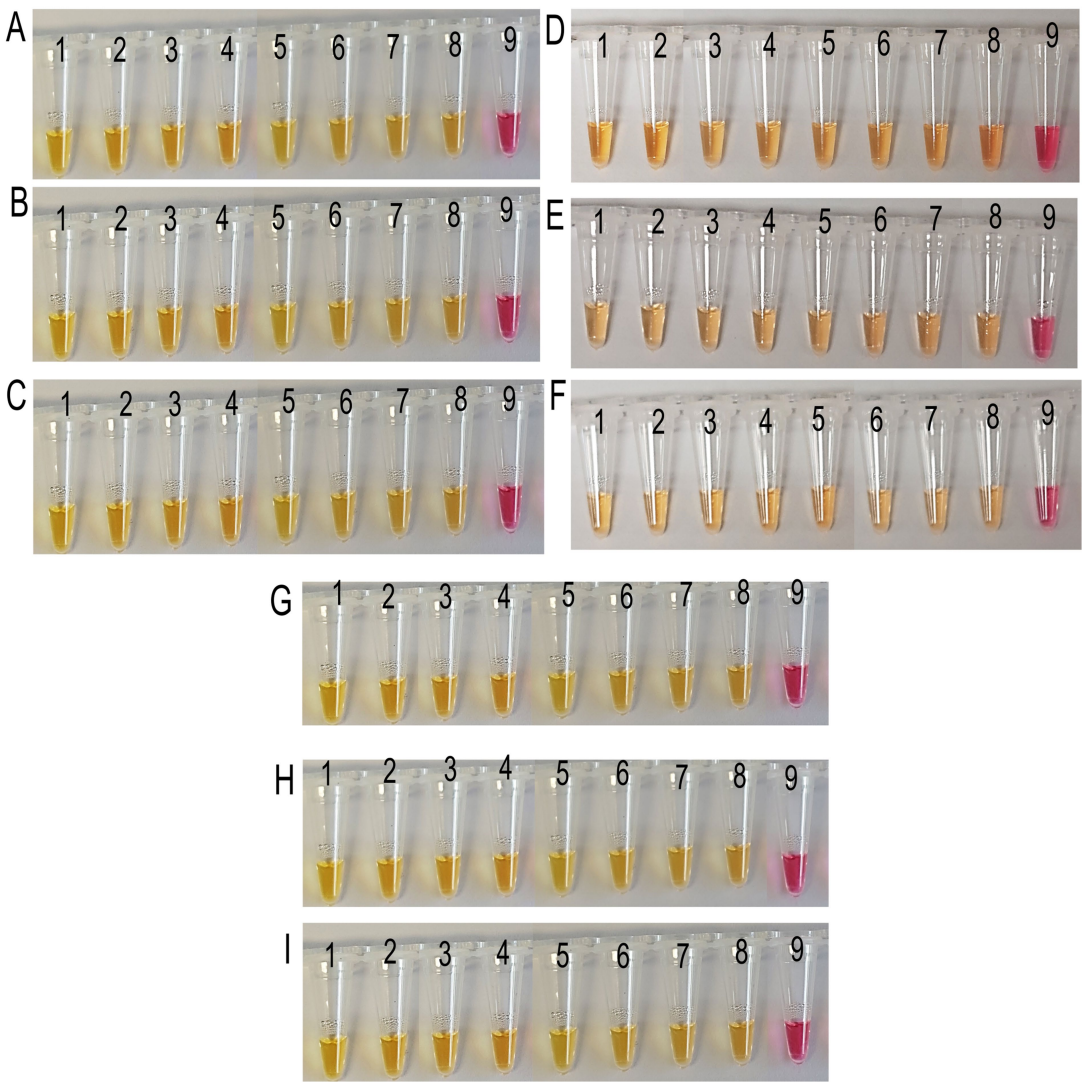
Field application of LAMP colorimetric assay for all the analyzed sugarcane samples collected from SRA Woodford LS screening trials. **(A–C)** Xylem sap, leaf tissue, and meristematic tissue samples collected in July 2022. **(D–F)** Xylem sap, leaf tissue, and meristematic tissue samples collected in October 2022. **(G–I)** Xylem sap, leaf tissue, and meristematic tissue samples collected in January 2023. Where, tubes 1 to 8: LS-infected xylem sap samples-Q87, Q63, Q68, Q208, Q96, Q124, Q44, and Q133; tube 9: no target control (NTC).

**Figure 5:**
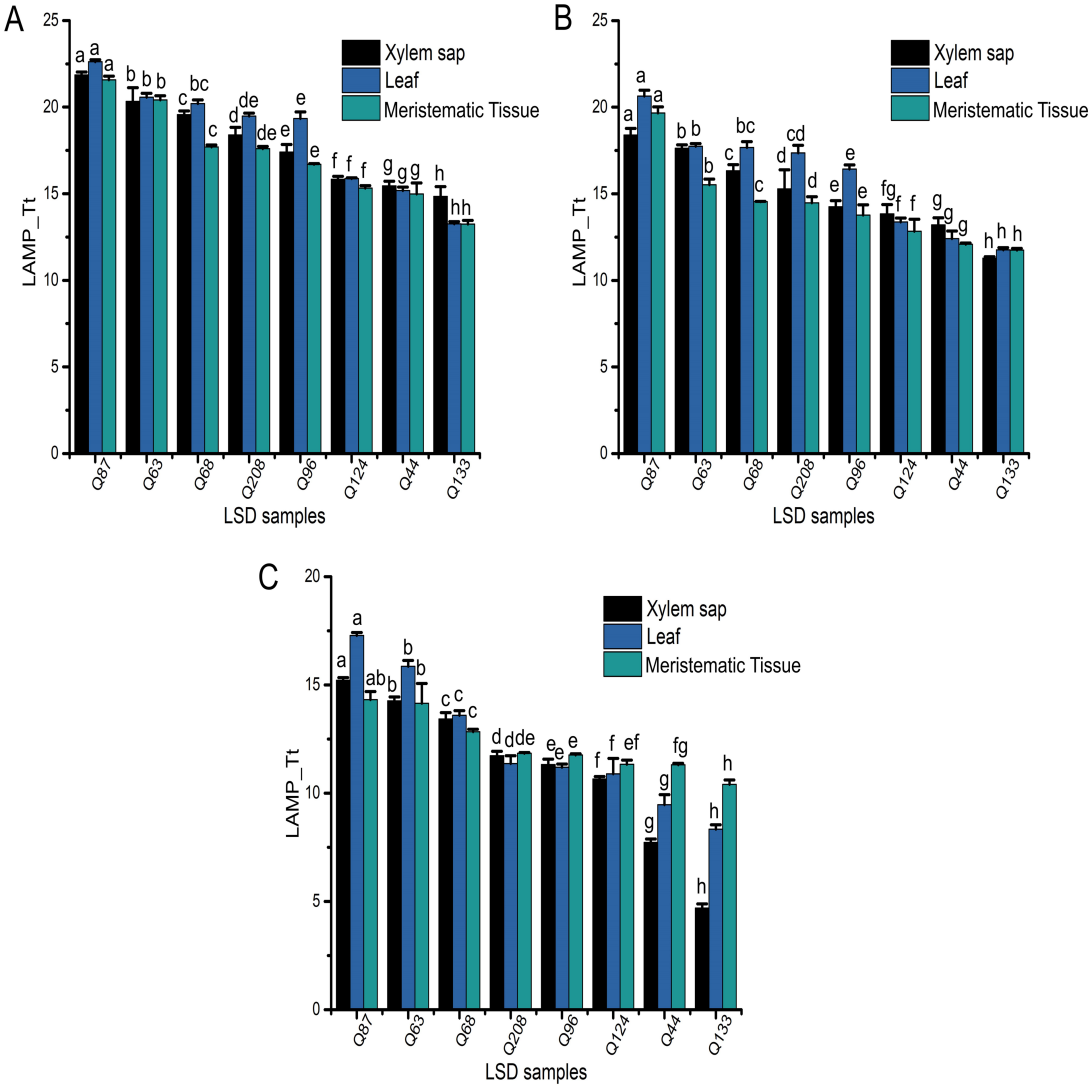
Field application of LAMP Fluorescence assay for all the analyzed field collected samples from the SRA Woodford LS trial sites. Obtained LAMP-Tt values of xylem sap, leaf tissue, and meristematic tissue samples collected in **(A)** July 2022, **(B)** October 2022, or **(C)** January 2023. Each bar is the mean of three replications, and bar associated with the same letter for a particular sample type is not significantly different according to Fisher’s protected LSD test (*p* = 0.05).

Target DNA was amplified from all the field samples collected from the first LS field trial, indicating varying levels of pathogen load (Figures 4, 5). In colorimetric LAMP, a yellow color development was observed in all field samples, providing a rapid and consistent qualitative indication of bacteria in the sample (Figure 4).

The fluorescent LAMP assay effectively amplified *Xalb* DNA from all collected samples across different Tt values, reflecting varying levels of pathogen load (Figures 5A–C). Most samples exhibited *Xalb* DNA concentrations within the assay’s lowest detection limit of 1 aM, highlighting its suitability for practical field applications. The detected *Xalb* DNA level in the different sugarcane samples correlated with bacterial load. For example, the Q87, Q63, Q68, and Q208 cultivars showed relatively higher Tt values (11.36–22.62) for all the analyzed sample types collected at different time points, suggesting lower *Xalb* presence (approximately 10^4^–10^7^ cells/μL) in xylem sap, leaf tissue, and meristematic tissue (Figures 3B, 5A–C). Conversely, cultivars Q44, Q96, Q124, and Q133 displayed progressively lower Tt values (4.69–19.33), indicating higher bacterial load (around 10^7^–10^9^ cells/μL) in these samples (Figures 3B, 5A–C). This data demonstrates that the LAMP method is effective for detecting and quantifying *Xalb* titres in infected sugarcane samples across various cultivars, irrespective of their resistance status. Notably, significantly different amounts of the amplified product of expected size (196 bp) for *Xalb* DNA were detected among them, with no amplification observed in control reactions (Supplementary Figure S4).

**Figure 6:**
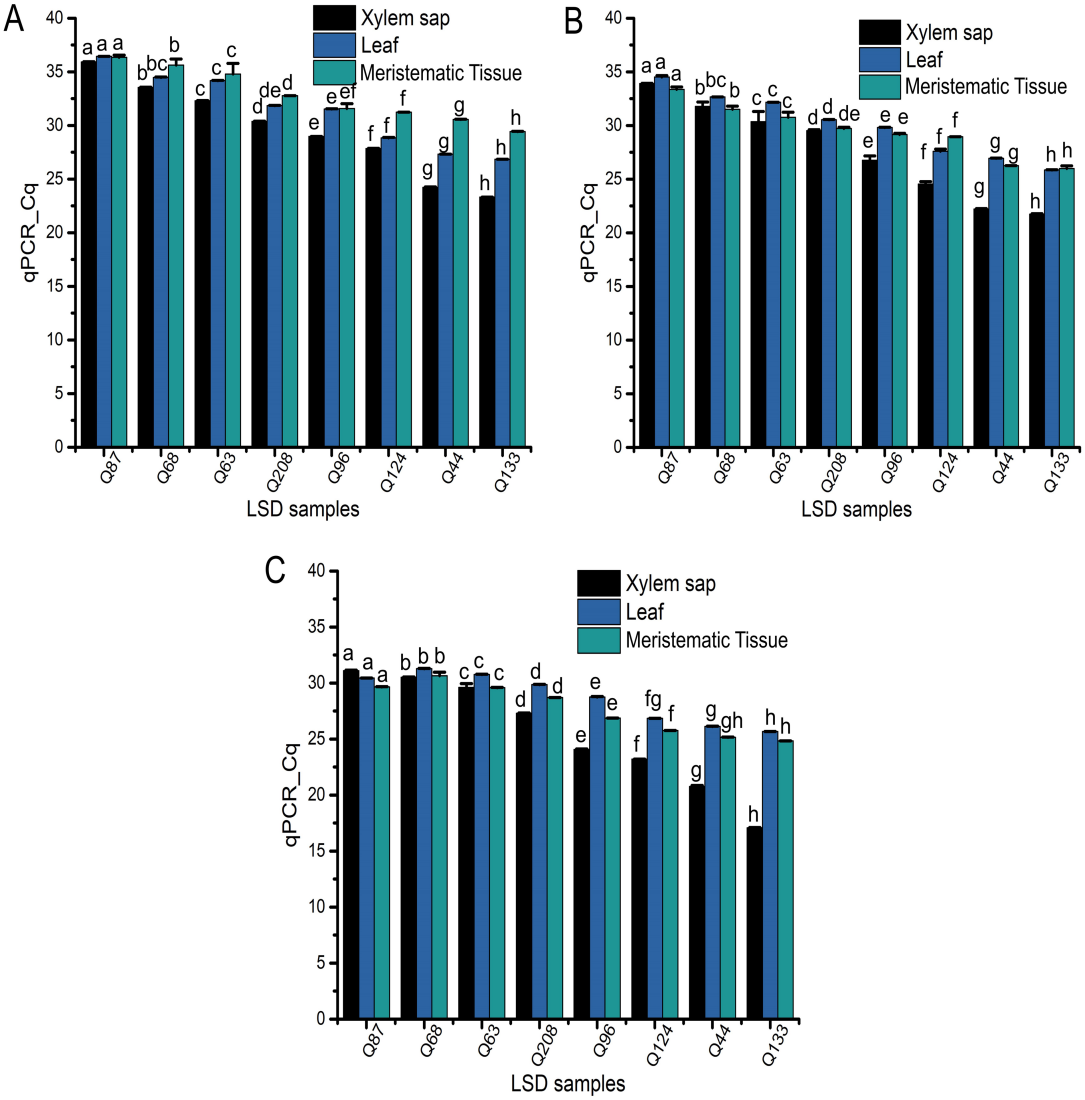
Field application of qPCR assay for all the analyzed sugarcane samples collected from the SRA Woodford LS screening trials. Obtained qPCR-Cq values of xylem sap, leaf tissue, and meristematic tissue samples collected in **(A)** July 2022, **(B)** October 2022, or **(C)** January 2023. Each bar is the mean of three replications, and bar associated with same letter for a particular sample type is not significantly different according to Fisher’s protected LSD test (*p* = 0.05).

The target qPCR region was successfully amplified from all infected field samples (Figure 6), revealing a significant correlation (*r* = 0.9, *p* < 0.001) between the qPCR values and the results of the LAMP assay, as outlined in Table 3. Despite the differences in amplification properties between LAMP and qPCR, strong correlations (*r* > 0.9) were observed between LAMP and qPCR results (Table 3), regardless of sample collection date or site. Additionally, a single amplicon was observed on the gel from positive samples only (Supplementary Figure S7), confirming the specificity of the assay.

**Table 3:** Spearman correlation coefficients determined between LAMP and qPCR data collected from different parts of sugarcane plants and at three different post-inoculation collection time points.

Sample origin	July 2022	October 2022	January 2023
Xyem sap	0.97***	0.97***	0.98***
Meristematic tissue	0.96***	0.94***	0.91***
Leaf tissue	0.98***	0.93***	0.95***

Correlations were based on 48 observations. *** = significant at or <0.001.

**Figure 7:**
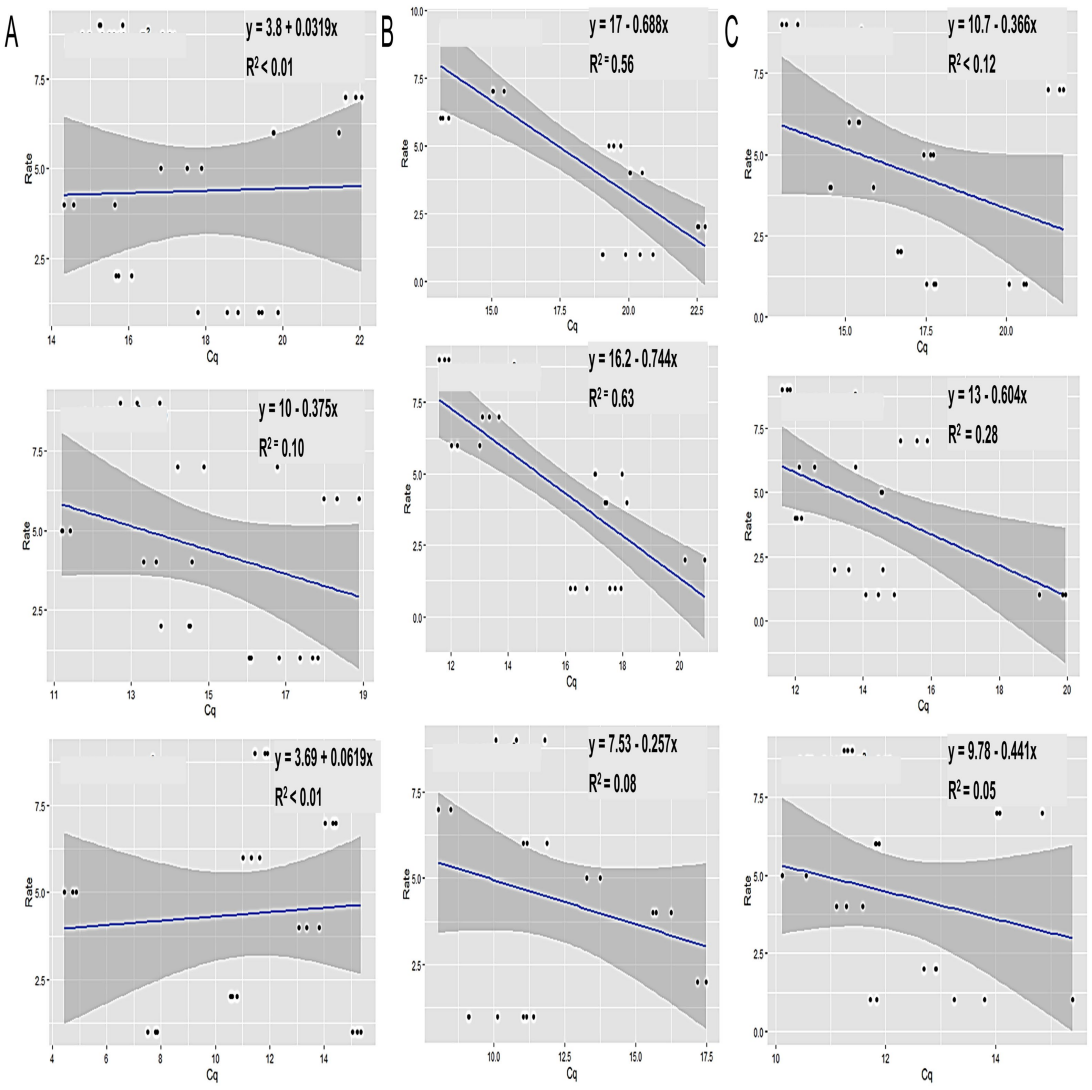
The relationship between LS disease ratings (rate) and Tt values. **(A)** Xylem sap, **(B)** leaf tissue, and **(C)** meristematic tissue samples collected from sugarcane cultivars with various scales of disease rating (1 to 9), inoculated by decapitating and sprayed with *Xalb* after planting. Samples were taken at 5 (top), 8 (middle), and 11 (bottom) months after inoculation, respectively.

When analyzing the Tt values from the LAMP experiment across different sample types using the SAS Proc Mixed Model, significant differences (*p* < 0.0001) were observed in Tt values (indicating *Xalb* load) among clones, sample origins (leaf tissue, meristematic tissue, or xylem sap), sampling times, or their interactions (Supplementary Table S1). Strong correlations were identified between leaf tissue samples and disease ratings when collected at 5 months (*r* = 0.75) and 8 months (*r* = 0.8) post-inoculation. However, no differences in Tt values were found between cultivars when samples were taken 11 months after inoculation (Figure 7A). A moderate correlation (*r* = 0.53) was observed between varietal resistance and Tt values at around 8 months post-inoculation, while low or no correlation was found at 5- or 11 months post-inoculation (Figure 7B). Additionally, there was no meaningful relationship between varietal resistance and Tt values in xylem sap (Figure 7C). These results suggest that leaves may be the suitable plant part for effective sampling for leaf scald diagnosis and varietal disease rating.

### 3.4 Identification of the most suitable leaf/leaves tissue for sampling

Significant differences were observed in Tt and Cq values among cultivars collected from the second LS trial and between the methods used (LAMP and qPCR). Specifically, in the interaction between cultivar and method (*p* < 0.001) and between the two methods themselves (*p* < 0.001) (Table 4). However, no significant differences were found between leaf locations within a variety, indicating a similar *Xalb* load among the leaf positions (TVD, TVD +1 and TVD −1) on a sugarcane stalk (Table 4).

**Table 4:** Type 3 test of fixed effects of cultivar and interactions.

Effect	Numerator DF	Denominator DF	*F* value	*Pr* > *F*
Cultivar	9	1,380	220.61	<2.2 × 10^−16^***
Leaf	2	1,380	9.52	7.8 × 10^−5^
Method	1	1,380	2135.11	<2.2 × 10^−16^***
Cultivar × Leaf	18	1,380	0.21	0.9998
Cultivar × Method	9	1,380	7.18	3.1 × 10^−10^***
Leaf × Method	2	1,380	0.23	0.7952
Cultivar × Leaf × Method	18	1,380	0.20	0.9999

*** = significant at or <0.001 levels.

### 3.5 Development of *Xalb* populations in various sugarcane samples over time

**Figure 8:**
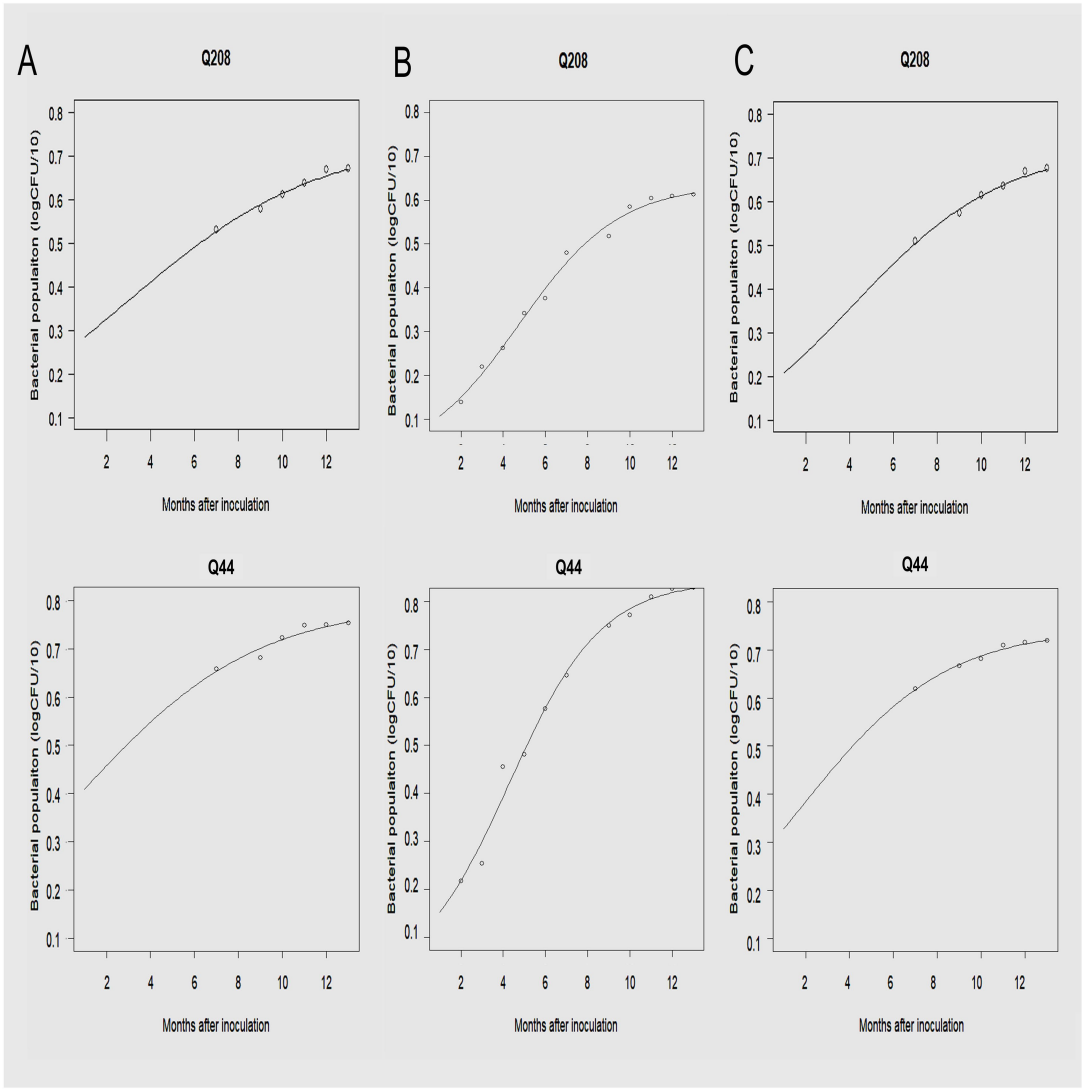
The development of *Xalb* population on a resistant (Q208) and a susceptible (Q44) cultivar in **(A)** xylem sap, **(B)** leaf tissue, and **(C)** meristematic tissue of sugarcane plants over time.

Following inoculation, the development of the *Xalb* population over time in leaf tissue, xylem, and meristematic tissues were analyzed for two sugarcane cultivars collected from the third LS trial, described by the regression equation in Table 5 and illustrated in Figure 8. The initial *Xalb* population, as estimated from the regression equations, was approximately 2.4 × 10^3^ Cells/μL for the susceptible cultivar Q44 and less than 60 Cells/μL for the resistant cultivar Q208. The maximum *Xalb* levels in the leaf tissue of Q208 and Q44 were estimated to be approximately 1.1 × 10^8^ Cells/μL and 1.8 × 10^9^ Cells/μL, respectively, indicating that the levels in Q44 were more than 17 times higher than those in Q208. Indeed, the rate of *Xalb* reproduction was slower in Q208 compared to Q44; taking about 146 days for Q208 to reach 50% of its maximum Cells, while Q44 reached it in approximately 131 days. Similar trends were observed among the meristematic tissues and xylem saps between the two cultivars.

**Table 5:** Regression equation describing *Xalb* population development over time in leaf tissue, meristematic tissue, and xylem sap samples.

Cultivar/Sample types	Equation	*R* ^2^
Q208		
Leaf tissue	0.634/(1 + *e*^−0.42(*t*−4.76)^)	0.98
Meristematic tissue	0.722/(1 + *e*^−0.29(*t*−4.14)^)	0.94
Xylem sap	0.736/(1 + *e*^−0.23(*t*−2.98)^)	0.94
Q44		
Leaf tissue	0.843/(1 + *e*^−0.46(*t*−4.29)^)	0.98
Meristematic tissue	0.744/(1 + *e*^−30(*t*−1.79)^)	0.88
Xylem sap	0.792/(1 + *e*^−0.25(*t*−0.73)^)	0.77

## 4 Discussion

This proof-of-concept study developed a highly specific and sensitive LAMP detection method for the quantification of *Xanthomonas albilineans* (*Xalb*) in sugarcane. The amplification mechanism of LAMP differs from qPCR by operating isothermally without discrete cycles. Instead, LAMP uses threshold time (Tt), the point at which fluorescence exceeds a set threshold, to quantify template concentration (Schmidt et al., 2021). Our study shows that Tt reliably correlates with initial template concentration under optimized conditions (Njiru et al., 2008), similar to the cycle quantification threshold (Cq) of qPCR. Previous LAMP and PCR protocols described in the literature targeted the ITS region, the *albI* gene, and the XAF DNA fragment. However, these methods often amplified anonymous fragments from *Xalb* genomic DNA (Dias et al., 2018; Garces et al., 2014; Gutierrez et al., 2016; Jensen et al., 1993; Wang et al., 1999; Welsh and McClelland, 1991). Existing primers (L1/G1, T3A/T5A, XAF1/XAR1, XAprF6/XAprR6, and FIP/BIP, F3/B3) were found to lack specificity for *Xalb*, as they also detected saprophytic bacteria from sugarcane (Pieretti et al., 2015). To address this limitation, we designed primers (Table 2) targeting the *XALB1* albicidin pathotoxin biosynthesis gene cluster, optimizing specificity and efficiency. Primer efficiency was assessed based on the intensity of amplification products of the expected and specific 196-bp target bands (Supplementary Figures S3, S4). The specificity of three primer pairs was further validated by testing their ability to detect *Xalb* in the *Saccharum* genus and other sugarcane pathogens such as *Lxx* (Figure 3; Supplementary Figure S3). Results confirmed the strong specificity of the designed primers, as they consistently produced specific amplification products (196 bp). However, additional testing with related pathogens and sugarcane isolates is necessary to ensure the LAMP assay’s efficacy across other *Xalb* serogroups or haplotypes.

A near-ideal amplification efficiency (slope ≈ 3) observed in the florescence LAMP standard curve (Figures 2B, 3B) which reflects the high reproducibility of the assay under optimized conditions. Although LAMP operates differently from qPCR, continuous amplification can produce a log-linear relationship when fluorescence thresholds are consistently defined. The efficiency highlights the optimized conditions, such as precise temperature control, primer design, and consistent fluorescence thresholding, supporting LAMP’s potential for quantitative applications (Mori et al., 2004). Our LAMP assay demonstrated higher sensitivity than conventional qPCR, detecting *Xalb* at 100-fold lower cell concentrations compared to qPCR. This sensitivity surpasses previous reports of *Xalb* detection by LAMP using purified DNA (10 Cells/mL) (Dias et al., 2018). While qPCR and nested-PCR methods have been shown to achieve low detection limits (10^2^–10^3^ Cells) for *Xanthomonas albilineans*, they are limited in their ability to determine pathogen population densities directly (Garces et al., 2014; Dias et al., 2018; Wang et al., 2020). Additionally, qPCR requires sophisticated and costly equipment, as well as labor-intensive sample preparation to mitigate reaction inhibition from contaminants. Nested PCR involves an extra gel electrophoresis step, adding to the complexity and time required. In contrast, this study highlights the compatibility of a reagent-free, heat-induced DNA extraction method with LAMP, enabling a simple, rapid, and visually detectable diagnostic solution with quasi-quantitative capabilities for *Xalb*. This builds on prior findings that bacterial cells are vulnerable to high-temperature damage (Jose and Brahmadathan, 2006; Umer et al., 2021). By eliminating the need for multistep sample processing and expensive commercial kits, our approach offers a cost-effective and accessible alternative, suitable for practical applications in field settings.

One of the challenging aspects of leaf scald disease (LS) is that many sugarcane plants infected with *Xalb* do not exhibit symptoms (Rott et al., 1997; Gutierrez et al., 2016), or the symptoms are so subtle that they escape detection (Rott et al., 1988; Ricaud and Ryan, 1989). In this study, we evaluated the detection efficiency of our assay by analyzing various sugarcane samples (leaf tissue, meristematic tissue, and xylem sap) collected from 10 cultivars 2–3 months post-inoculation. The successful detection of *Xalb* in field-derived samples from asymptomatic stalks confirmed the high efficiency of our assay for identifying *Xalb* in contaminated field samples, as previously demonstrated for LAMP (Dias et al., 2018). Our findings showed that bacterial populations in leaf tissue are a more reliable indicator of the genetic resistance of sugarcane varieties to LS compared to other sample types, which aligns with traditional assessments of LS resistance based on disease incidence and severity. A strong correlation between *Xalb* populations in the shoot apex and disease severity, as reported by Rott et al. (1997), underscores the value of accurate bacterial quantification for resistance screening. Traditionally, resistance screening has relied on the erratic expression of symptoms following inoculation (Rott and Davis, 2000). This study demonstrated that LAMP-based quantification of *Xalb* can distinguish differences among sugarcane cultivars with varying levels of resistance to LS by analyzing leaf tissues. Consistent with previous findings (Garces et al., 2014; Gutierrez et al., 2016), young, systemically infected leaves proved to be the best tissue for detecting differences between clones. Regression analysis of bacterial population data over time indicated that Cell counts from leaf tissue provided a significantly better fit for the model compared to other sample types (Figure 7). This suggests that leaf tissue is the most suitable sample for consistent quantification of *Xalb* populations using LAMP. Moreover, leaf sampling is straightforward, minimally invasive, and does not require specialized DNA extraction methods or kits.

Additionally, our results showed that LS bacteria proliferate at higher rates in susceptible varieties compared to resistant ones. This supports earlier work by Rott et al. (1997), which demonstrated that sugarcane variety resistance is correlated with bacterial populations in the apex. Subsequent studies by Garces et al. (2014) and Gutierrez et al. (2016) also confirmed that quantifying *Xalb* in sugarcane clones using qPCR can effectively differentiate resistance categories. For the first time, we successfully applied the LAMP method to distinguish between susceptible and resistant sugarcane varieties. Moving forward, we plan to expand our testing to include additional sugarcane clones with varying resistance levels to further validate the LAMP method.

To the best of our knowledge, this study is the first to demonstrate the use of LAMP for molecular detection and quantification of *Xalb*, and evaluation of leaf scald (LS) resistance levels. Accurate determination of LS resistance in sugarcane clones is critical for breeding programs, but the variable and erratic expression of disease symptoms under natural conditions or after inoculation makes this process challenging. Previous research highlighted the potential of quantifying *Xalb* populations with qPCR for resistance evaluation (Garces et al., 2014). Our results confirmed that bacterial quantification is more reliable than assessing visual symptom severity across cultivars with varying resistance levels. This study demonstrated that LAMP offers an improved method for evaluating LS resistance during screening and resistance studies. The simplicity of this newly developed LAMP assay eliminates the need for sophisticated equipment, specialized reagents, or advanced laboratory infrastructure, making it highly suitable for use in under-resourced settings. The method requires minimal training and delivers results within 40 min. Additionally, the heat-based DNA extraction method provides a cost-effective and convenient alternative to traditional approaches. Its compatibility with LAMP offers potential for field deployment, especially when integrated with handheld devices, enabling on-site analysis of multiple stalks and cultivars during commercial screening procedures. While promising, further experiments are needed to refine the method and ensure consistent and accurate determination of clone resistance levels to LS using LAMP.

## 5 Conclusion

In summary, this study has successfully reported a reagent-free DNA isolation method as a proof-of-concept for the molecular detection of the LS disease-causing pathogen *Xalb* in sugarcane leaf tissue, meristematic tissue, and sap samples. This innovative assay combines heat-induced *Xalb* cell lysis, the release of genetic contents, and the targeted DNA region amplification using LAMP techniques. Our method offers a dual detection capability, where qualitative detection of *Xalb* is achieved through naked-eye evaluation of color change, while fluorescence signals allow for quantification up to femtomolar levels of target DNA. The assay exhibits high specificity, a good linear response (*r* = 0.99) for detecting target concentrations from 10 pM to 1 aM, and excellent repeatability (SD <5%, *n* = 3). Furthermore, the practical applicability of this assay has been demonstrated through successful *Xalb* detection from field samples. We believe this method offers a promising approach for diagnosing and managing diseases caused by bacterial pathogens not only in sugarcane but also in other agricultural and horticultural crops. Moreover, this study holds the potential to be integrated with a portable device, incorporating a microcentrifuge tube-like microreactor made of a suitable material [e.g., polydimethylsiloxane (PDMS)], coupled with a distinct functional module for temperature control (heat). Such a device could enable *in-situ* isolation and quantification of *Xalb* DNA, making it a valuable tool for on-site applications. Furthermore, the LAMP method can be utilized to screen sugarcane clones at various stages of the selection program. However, further studies with a larger number of sugarcane clones are needed to validate the reliability and repeatability of this method.

## Data Availability

The original contributions presented in the study are included in the article/Supplementary material, further inquiries can be directed to the corresponding author.

## Footnotes

^1^ www.snapgene.com

## References

[ref1] BioRender. (2022). Available at: https://www.biorender.com/ (Accessed September 20, 2024)

[ref2] Birch R. G. (2001). *Xanthomonas albilineans* and the antipathogenesis approach to disease control. Mol. Plant Pathol. 2, 1–11. doi: 10.1046/j.1364-3703.2001.00046.x, PMID: 20572987

[ref3] Birch R. G. Patil S. S. (1985). Preliminary characterization of an antibiotic produced by *Xanthomonas albilineans* which inhibits DNA synthesis in *Escherichia coli*. Microbiology 131, 1069–1075. doi: 10.1099/00221287-131-5-1069, PMID: 2410547

[ref4] Chakraborty M. Bhuiyan S. A. Strachan S. Saha S. Mahmudunnabi R. G. Nguyen N.-T.. (2024). A novel *Leifsonia xyli* subsp. *xyli* quantitative LAMP-based diagnostic correlated with sugarcane ratoon stunting disease rating. Crop Pasture Sci. 75:CP24053. doi: 10.1071/CP24053

[ref5] Chen Y. Ye W. Zhang Y. Xu Y. (2015). High-speed BLASTN: an accelerated MegaBLAST search tool. Nucleic Acids Res. 43, 7762–7768. doi: 10.1093/nar/gkv784, PMID: 26250111 PMC4652774

[ref6] Cociancich S. Pesic A. Petras D. Uhlmann S. Kretz J. Schubert V.. (2015). The gyrase inhibitor albicidin consists of *p*-aminobenzoic acids and cyanoalanine. Nat. Chem. Biol. 11, 195–197. doi: 10.1038/nchembio.1734, PMID: 25599532

[ref7] Croft B. Greet A. Leaman T. Teakle D. (1994). RSD diagnosis and varietal resistance screening in sugarcane using the EB-EIA technique. Proc. Aust. Soc. Sugar Cane Technol. 3, 143–151.

[ref8] Davis M. Dean J. Harrison N. (1988). Quantitative variability of *Clavibacter xyli* subsp. *xyli* populations in sugarcane cultivars differing in resistance to ratoon stunting disease. Phytopathology 78, 462–468. doi: 10.1094/Phyto-78-462

[ref9] Davis M. J. Gillaspie A. G. Jr. Harris R. W. Lawson R. H. (1980). Ratoon stunting disease of sugarcane: isolation of the causal bacterium. Science 210, 1365–1367. doi: 10.1126/science.210.4476.1365, PMID: 17817853

[ref10] Davis M. J. Rott P. Baudin P. Dean J. L. (1994). Evaluation of selective media and immunoassays for detection of *Xanthomonas albilineans*, causal agent of sugarcane LS disease. Plant Dis. 78, 78–82. doi: 10.1094/PD-78-0078

[ref11] Davis M. J. Rott P. Warmuth C. Chatenet M. Baudin P. (1997). Intraspecific genomic variation within *Xanthomonas albilineans*, the sugarcane LS pathogen. Phytopathology 87, 316–324. doi: 10.1094/PHYTO.1997.87.3.316 18945175

[ref12] Dias D. V. Fernandez E. Cunha M. G. Pieretti I. Hincapie M. Roumagnac P.. (2018). Comparison of loop-mediated isothermal amplification, polymerase chain reaction, and selective isolation assays for detection of *Xanthomonas albilineans* from sugarcane. Trop. Plant Pathol. 43, 351–359. doi: 10.1007/s40858-018-0216-2

[ref13] Francois P. Tangomo M. Hibbs J. Bonetti E. J. Boehme C. C. Notomi T.. (2011). Robustness of a loop-mediated isothermal amplification reaction for diagnostic applications. FEMS Immunol. Med. Microbiol. 62, 41–48. doi: 10.1111/j.1574-695X.2011.00785.x, PMID: 21276085

[ref14] Garces F. F. Gutierrez A. Hoy J. W. (2014). Detection and quantification of *Xanthomonas albilineans* by qPCR and potential characterization of sugarcane resistance to LS. Plant Dis. 98, 121–126. doi: 10.1094/PDIS-04-13-0431-RE, PMID: 30708616

[ref15] Gutierrez A. Garces F. F. Hoy J. W. (2016). Evaluation of resistance to leaf scald by quantitative PCR of *Xanthomonas albilineans* in sugarcane. Plant Dis. 100, 1331–1338. doi: 10.1094/PDIS-09-15-1111-RE 30686195

[ref16] Jensen M. A. Webster J. A. Straus N. (1993). Rapid identification of bacteria on the basis of polymerase chain reaction-amplified ribosomal DNA spacer polymorphisms. Appl. Environ. Microbiol. 59, 945–952. doi: 10.1128/aem.59.4.945-952.1993, PMID: 8476298 PMC202221

[ref17] Jose J. Brahmadathan K. (2006). Evaluation of simplified DNA extraction methods for *emm* typing of group a streptococci. Indian J. Med. Microbiol. 24, 127–130. doi: 10.1016/S0255-0857(21)02413-0, PMID: 16687865

[ref18] Koike H. (1965). The aluminium-cap method for testing sugarcane varieties against leaf scald disease. Phytopathology 55:317.

[ref19] Lin L. H. Ntambo M. S. Rott P. C. Wang Q. N. Lin Y. H. Fu H. Y.. (2018). Molecular detection and prevalence of *Xanthomonas albilineans*, the causal agent of sugarcane LS, in China. Crop Prot. 109, 17–23. doi: 10.1016/J.CROPRO.2018.02.027

[ref20] Mensi I. Vernerey M. S. Gargani D. Nicole M. Rott P. (2014). Breaking dogmas: the plant vascular pathogen *Xanthomonas albilineans* is able to invade non-vascular tissues despite its reduced genome. Open Biol. 4:130116. doi: 10.1098/rsob.130116, PMID: 24522883 PMC3938051

[ref21] Mori Y. Kitao M. Tomita N. Notomi T. (2004). Real-time turbidimetry of LAMP reaction for quantifying template DNA. J. Biochem. Biophys. Methods 59, 145–157. doi: 10.1016/j.jbbm.2003.12.005, PMID: 15163526

[ref22] National Center for Biotechnology Information (1988). *Leifsonia xyli* subsp. *xyli* str. CTCB07, complete genome. Accession No. AE016822. Bethesda, MD: National Library of Medicine (US), NCBI.

[ref23] Njiru Z. K. Mikosza A. S. J. Armstrong T. Enyaru J. C. Ndung'u J. M. Thompson A. R. C. (2008). Loop-mediated isothermal amplification (LAMP) method for rapid detection of *Trypanosoma brucei rhodesiense*. PLoS Negl. Trop. Dis. 2:e147. doi: 10.1371/journal.pntd.0000147, PMID: 18253475 PMC2238707

[ref24] Ntambo M. S. Meng J. Y. Rott P. C. Royer M. Lin L. H. Zhang H. L.. (2019). Identification and characterization of *Xanthomonas albilineans* causing sugarcane LS in China using multilocus sequence analysis. Plant Pathol. 68, 269–277. doi: 10.1111/ppa.12951

[ref25] Pan Y.-B. Grisham M. P. Burner D. M. Legendre B. L. Wei Q. (1999). Development of polymerase chain reaction primers highly specific for *Xanthomonas albilineans*, the causal bacterium of sugarcane LS disease. Plant Dis. 83, 218–222. doi: 10.1094/PDIS.1999.83.3.218, PMID: 30845497

[ref26] Pieretti I. Cociancich S. Bolot S. Carrere S. Morisset A. Rott P.. (2015). Full genome sequence analysis of two isolates reveals a novel *Xanthomonas* species close to the sugarcane pathogen *Xanthomonas albilineans*. Genes 6, 714–733. doi: 10.3390/genes6030714, PMID: 26213974 PMC4584326

[ref27] Pieretti I. Royer M. Barbe V. Carrere S. Koebnik R. Cociancich S.. (2009). The complete genome sequence of *Xanthomonas albilineans* provides new insights into the reductive genome evolution of the xylem-limited *Xanthomonadaceae*. BMC Genomics 10:616. doi: 10.1186/1471-2164-10-616 20017926 PMC2810307

[ref28] Plant Health Australia (2019). The National Plant Biosecurity Status Report 2018 and 2019. Canberra, ACT: Plant Health Australia.

[ref29] Ricaud C. Ryan C. C. (1989). “Leaf scald” in Diseases of sugarcane: major diseases. eds. Ricaud C. Egan B. T. Gillaspie A. G. Jr. Hughes C. G. (New York, NY: Elsevier), 39–58.

[ref30] Rott P. (1995). Leaf scald of sugarcane. Agric. Dev. 6, 49–56.

[ref31] Rott P. Chatenet M. Granier M. Baudin P. (1988). Leaf scald disease of sugarcane caused by *Xanthomonas albilineans* (Ashby) Dowson. II-three methods and host spectrum for diagnosis of the pathogen in tropical Africa. L’Agron. Trop. 43, 244–251.

[ref32] Rott P. Davis M.. (1995). Recent advances in research on variability of *Xanthomonas albilineans*, causal agent of sugarcane LS disease. Proceedings of the International Society of Sugarcane Technologists Congress. 498–503. International Society of Sugarcane Technologists.

[ref33] Rott P. Davis M. J. (2000). “Leaf scald” in A guide to sugarcane diseases. eds. Rott P. Bailey R. A. Comstock J. C. Croft B. J. Saumtally A. S. (Montpellier: CIRAD/ISSCT), 38–44.

[ref34] Rott P. Mohamed I. S. Klett P. Soupa D. de Saint-Albin A. Feldmann P.. (1997). Resistance to leaf scald disease is associated with limited colonization of sugarcane and wild relatives by *Xanthomonas albilineans*. Phytopathology 87, 1202–1213. doi: 10.1094/PHYTO.1997.87.12.1202, PMID: 18945019

[ref35] Royer M. Costet L. Vivien E. Bes M. Cousin A. Damais A.. (2004). Albicidin pathotoxin produced by *Xanthomonas albilineans* is encoded by three large PKS and NRPS genes present in a gene cluster also containing several putative modifying, regulatory, and resistance genes. Mol. Plant Microbe Interact. 17, 414–427. doi: 10.1094/MPMI.2004.17.4.414, PMID: 15077674

[ref36] Saxton A. M.. (1998). A macro for converting mean separation output to letter groupings in Proc Mixed. Proceedings of the 23rd SAS Users Group International. March 22–25, 1998. Nashville, TN. Cary, NC. SAS Institute. 1243–1246.

[ref37] Schmidt J. Berghaus S. Blessing F. Wenzel F. Herbeck H. Blessing J.. (2021). A semi-automated, isolation-free, high-throughput SARS-CoV-2 reverse transcriptase (RT) loop-mediated isothermal amplification (LAMP) test. Sci. Rep. 11:21385. doi: 10.1038/s41598-021-00827-0, PMID: 34725400 PMC8560768

[ref38] Tsai S. M. Chan K. W. Hsu W. L. Chang T. J. Wong M. L. Wang C. Y. (2009). Development of a loop-mediated isothermal amplification for rapid detection of ORF virus. J. Virol. Methods 157, 200–204. doi: 10.1016/j.jviromet.2009.01.003, PMID: 19186192

[ref39] Umer M. Aziz N. B. Al Jabri S. Bhuiyan S. A. Shiddiky M. J. A. (2021). Naked eye evaluation and quantitative detection of the sugarcane LS pathogen, *Xanthomonas albilineans*, in sugarcane xylem sap. Crop Pasture Sci. 72, 361–371. doi: 10.1071/CP20416

[ref40] Wang Z. K. Comstock J. C. Hatziloukas E. Schaad N. W. (1999). Comparison of PCR, BIO-PCR, DIA, ELISA, and isolation on semiselective medium for detection of *Xanthomonas albilineans*, the causal agent of LS of sugarcane. Plant Pathol. 48, 245–252. doi: 10.1046/j.1365-3059.1999.00332.x

[ref41] Wang H. B. Xiao N. Y. Wang Y. J. Guo J. L. Zhang J. S. (2020). Establishment of a qualitative PCR assay for the detection of *Xanthomonas albilineans* (Ashby) Dowson in sugarcane. Crop Prot. 130:105053. doi: 10.1016/j.cropro.2019.105053

[ref42] Welsh J. McClelland M. (1991). Genomic fingerprints produced by PCR with consensus tRNA gene primers. Nucleic Acids Res. 19, 861–866. doi: 10.1093/nar/19.4.861, PMID: 2017367 PMC333722

[ref43] Yang Z. Liu N. Y. Zhu Z. Xiao M. Zhong S. Xue Q.. (2022). Rapid and convenient detection of SARS-CoV-2 using a colorimetric triple-target reverse transcription loop-mediated isothermal amplification method. PeerJ 10:e14121. doi: 10.7717/peerj.14121, PMID: 36248705 PMC9558625

[ref44] Ye J. Coulouris G. Zaretskaya I. Cutcutache I. Rozen S. Madden T. L. (2012). Primer-BLAST: a tool to design target-specific primers for polymerase chain reaction. BMC Bioinform. 13, 1–11. doi: 10.1186/1471-2105-13-S6-S1, PMID: 22708584 PMC3412702

