# Supplementary material for: Development and validation of a LAMP-based method for rapid and reliable detection of Xanthomonas albilineans, the causal agent of sugarcane leaf scald
Source: Front Microbiol. 2025 Jan 30;16:1537812. doi: 10.3389/fmicb.2025.1537812 (PMC11821956; doi:10.3389/fmicb.2025.1537812)
Supplement: Supplementary file 1 [file Data_Sheet_1.docx]

Supplementary information

**Development and validation of a LAMP-based method for rapid and reliable detection of *Xanthomonas albilineans*, the causal agent of sugarcane leaf scald**


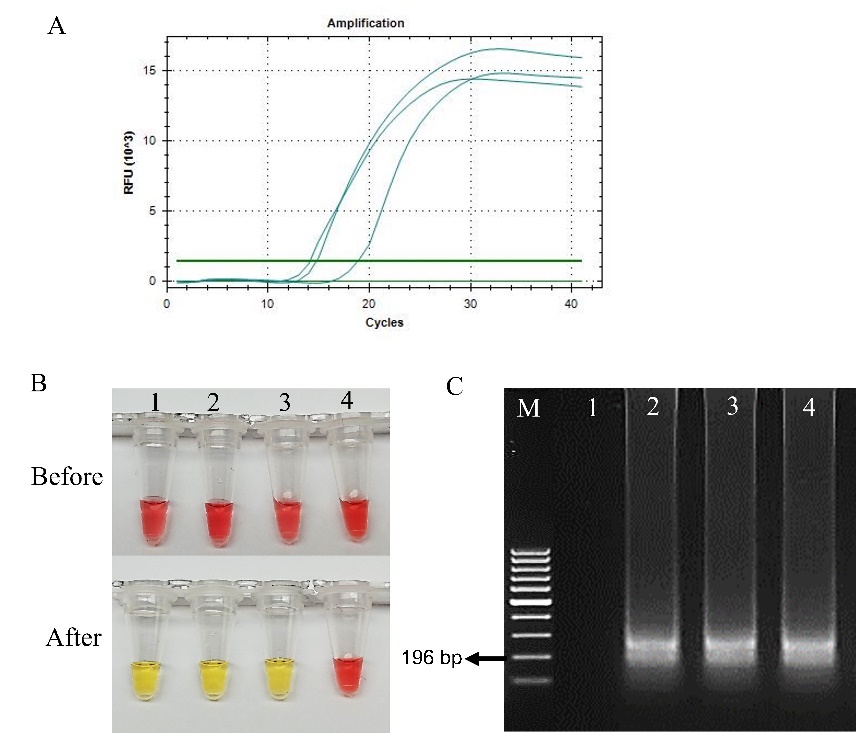


**Figure S1**. LAMP primer pairs checking with synthetic target. A) Amplification plot of serial dilutions of synthetic target; B) Fluorescent color change of LAMP amplified products. C) Agarose gel electropherograms of LAMP amplified products. Lane M: 100bp+ marker; Lanes 1 to 3: 10^7^ copies/𝜇L (10 pg/𝜇L) of synthetic target; Lane 4: No Target Control (NTC), (S/n = >3).


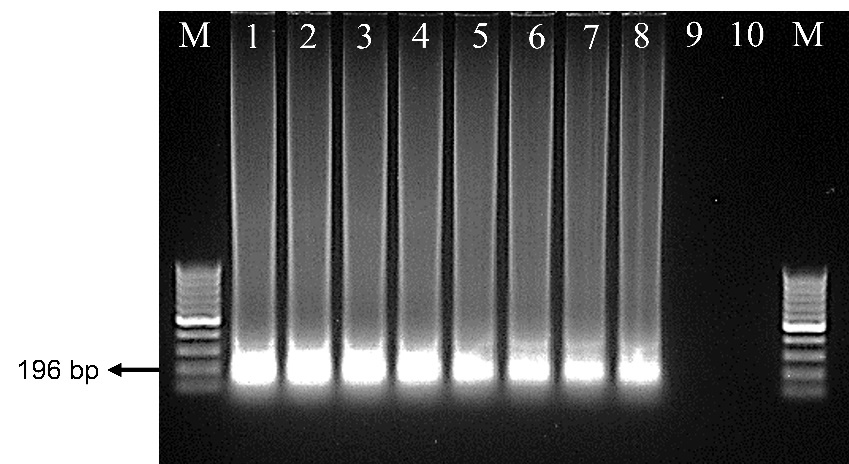


**Figure S2.** LAMP primer pairs checking with different synthetic target concentrations. Agarose gel electropherograms of LAMP amplified products. Lane M: 100bp+ marker; Lanes 1 to 8: 1:10 dilutions of synthetic target (10^7^-10^0^ copies/𝜇L, or 10 pg/𝜇L-1ag/𝜇L); Lane 9: No Target Control (NTC), (S/n = >3*).*


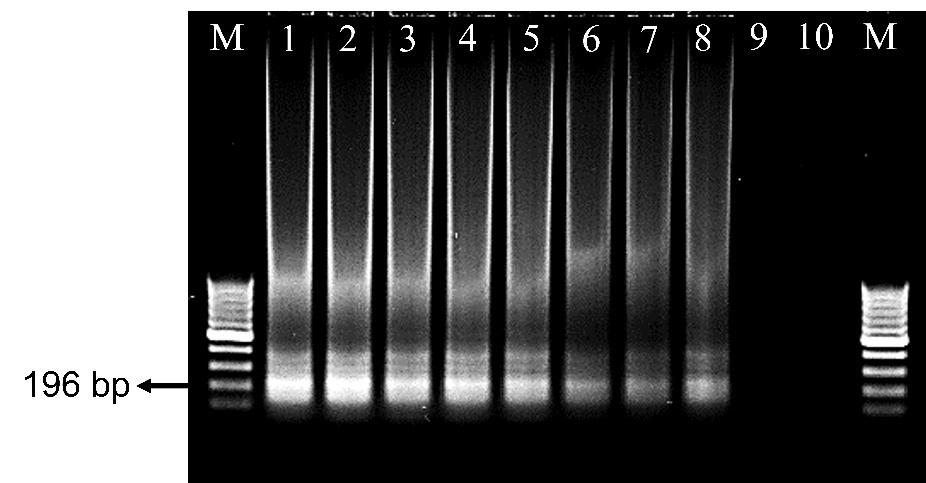


**Figure S3.** Sensitivity and specificity analysis. Agarose gel electropherograms of LAMP amplified products. Lane M: 100bp+ marker; Lanes 1 to 8: 1:10 dilutions of spiking cells (10^7^-10^0^ cells/𝜇L); Lane 9: No Target Control (NTC); Lane 10: Lxx cells (10^7^ cells/𝜇L). (S/n = >3).


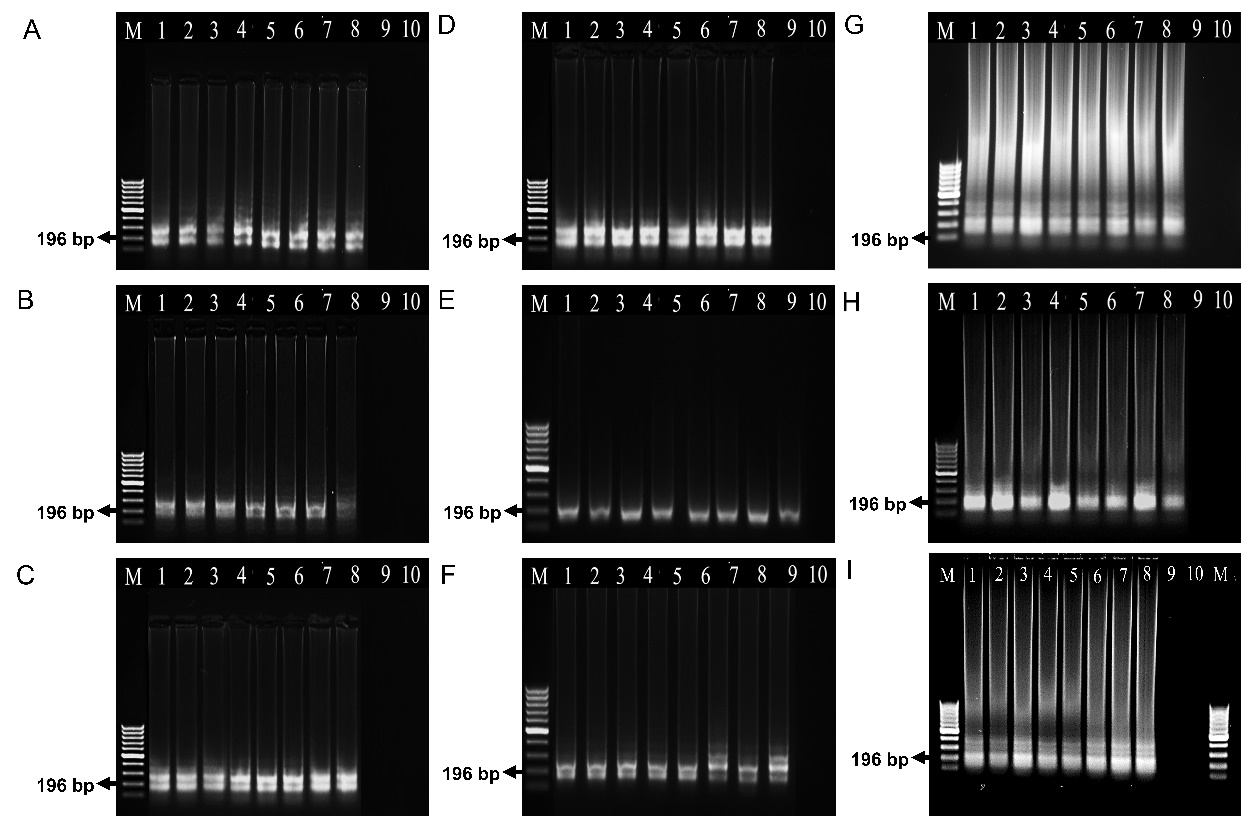


**Figure S4**. Field application of assay. Agarose gel electropherograms of LAMP amplified products. Lane M: 100bp+ marker; Lanes 1 to 8: LSD-infected xylem sap samples- Q133, Q124, Q44, Q63, Q87, Q68, Q296, and Q208; Lane 9: No Target Control (NTC); Lane 10: RSD-infected xylem sap sample. (S/n = >3).


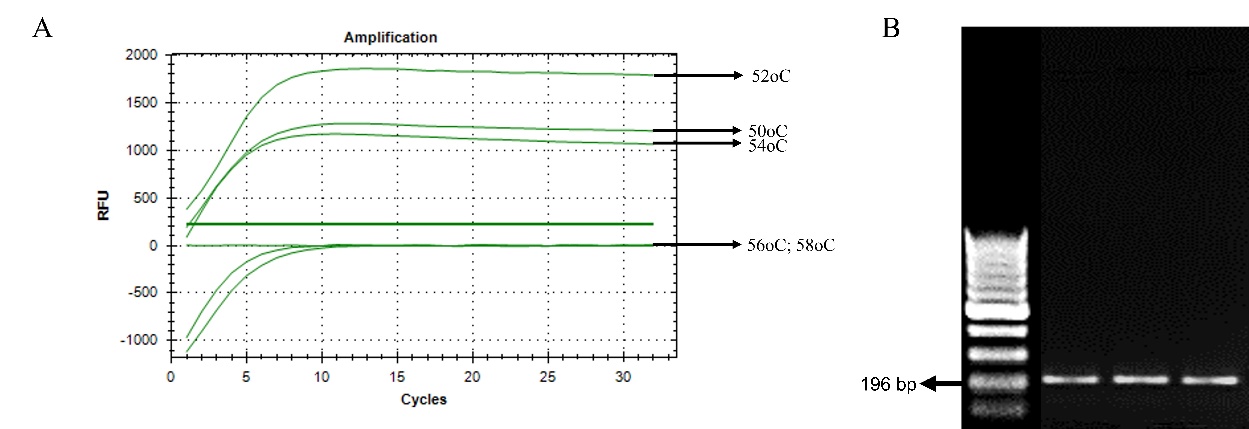


**Figure S5**. qPCR primer pair checking with synthetic target. A) Amplification plot of synthetic target; B) Agarose gel electropherograms of qPCR amplified products. Lane M: 100bp^+^ marker; Lanes 1 to 3: 10^10^ copies/𝜇L (10 ng/𝜇L) of synthetic target; Lane 4: No Target Control (NTC), (S/n = >3).

*
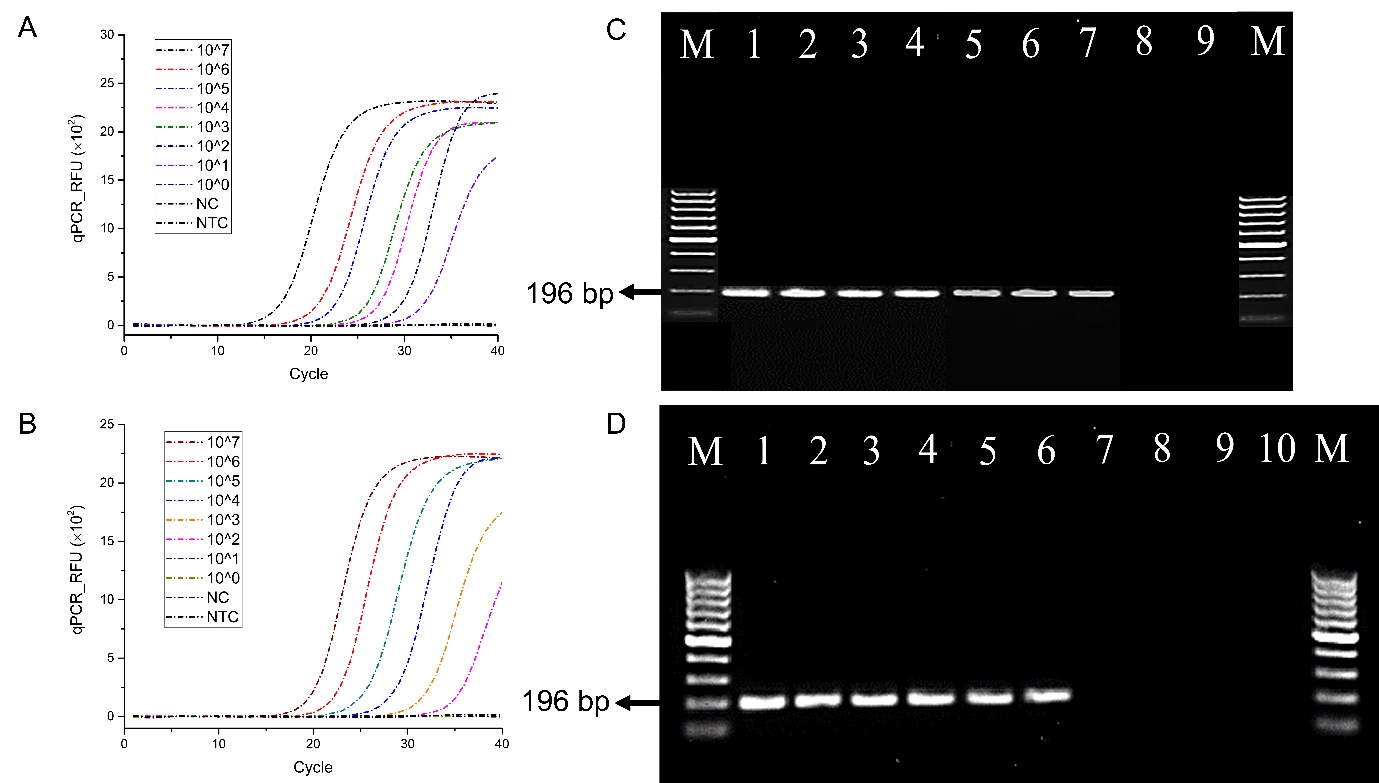
*

Figure S6. Validation of the assay using qPCR. Agarose gel electropherograms of qPCR amplified products. Lane M: 100bp+ marker; Lanes 1 to 8: Purified DNA extracted from known number of *Xalb* cells (10^7^-10^0^ cells/mL) spiking in the fresh sap; Lane 9: No Target Control (NTC); Lane 10: RSD-infected xylem sap sample. (S/n = >3).


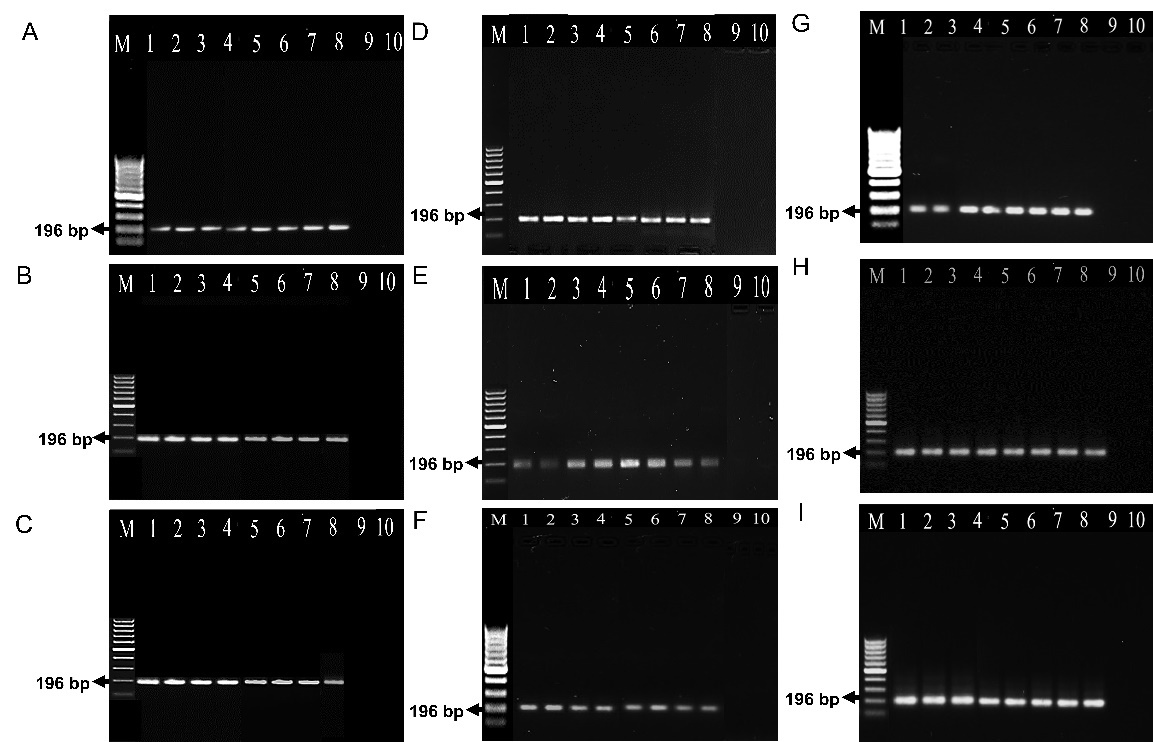


**Figure S7**: Field sample validation with qPCR. Fluorescence qPCR detection for all the analyzed xylem sap, leaf and meristematic tissue samples collected from SRA Woodford RSD screening trials; (A, B, C) Xylem sap, leaf, and meristematic tissue samples collected in July 2022; (D, E, F) Xylem sap, leaf, and meristematic tissue samples collected in October 2022; (G, H, I) Xylem sap, leaf, and meristematic tissue samples collected in January 2023. Agarose gel electrophoresis of qPCR amplified products. Where, Lane M: 100bp+ marker; Lanes 1 to 8: LSD-infected samples- Q87, Q63, Q68, Q208, Q96, Q124, Q44, and Q133; Lane 11: No Target Control (NTC).

Table S1. Type 3 test of fixed effects of cultivar, sample origins, sampling times, or their interactions.

| **Effect** | **Numerator DF** | **Denominator DF** | ***F* Value** | ***Pr > F*** |
| --- | --- | --- | --- | --- |
| **Clone** | 7 | 144 | 190.86 | <.0001 |
| **Origin (leaf, tissue, and xylem sap)** | 2 | 144 | 82.93 | <.0001 |
| **Month (July22, Oct22, Jan23)** | 2 | 144 | 3542.89 | <.0001 |
| **Origin*Clone** | 14 | 144 | 310.02 | <.0001 |
| **Month*Origin** | 4 | 144 | 78.37 | <.0001 |
| **Month*Origin*Clone** | 42 | 144 | 73.27 | <.0001 |

*** = significant at or <0.0001 levels.
